# Detection of colorectal‐cancer‐associated bacterial taxa in fecal samples using next‐generation sequencing and 19 newly established qPCR assays

**DOI:** 10.1002/1878-0261.13700

**Published:** 2024-07-06

**Authors:** Thulasika Senthakumaran, Tone M. Tannæs, Aina E. F. Moen, Stephan A. Brackmann, David Jahanlu, Trine B. Rounge, Vahid Bemanian, Hege S. Tunsjø

**Affiliations:** ^1^ Department of Life Sciences and Health Oslo Metropolitan University Norway; ^2^ Section for Clinical Molecular Biology (EpiGen) Akershus University Hospital Lørenskog Norway; ^3^ Department of Clinical Molecular Biology, Institute of Clinical Medicine University of Oslo Norway; ^4^ Department of Methods Development and Analytics Norwegian Institute of Public Health Oslo Norway; ^5^ Department of Gastroenterology, Division of Medicine Akershus University Hospital Lørenskog Norway; ^6^ Institute for Clinical Medicine University of Oslo Norway; ^7^ Department of Pharmacy, Centre for Bioinformatics University of Oslo Norway; ^8^ Department of Research Cancer Registry of Norway Oslo Norway; ^9^ Department of Pathology Akershus University Hospital Lørenskog Norway

**Keywords:** 16S rRNA amplicon sequencing, colorectal cancer, contaminant species, fecal microbiota, mucosal microbiota, qPCR

## Abstract

We have previously identified increased levels of distinct bacterial taxa within mucosal biopsies from colorectal cancer (CRC) patients. Following prior research, the aim of this study was to investigate the detection of the same CRC‐associated bacteria in fecal samples and to evaluate the suitability of fecal samples as a non‐invasive material for the detection of CRC‐associated bacteria. Next‐generation sequencing (NGS) of the 16S ribosomal RNA (rRNA) V4 region was performed to evaluate the detection of the CRC‐associated bacteria in the fecal microbiota of cancer patients, patients with adenomatous polyp and healthy controls. Furthermore, 19 novel species‐specific quantitative PCR (qPCR) assays were established to detect the CRC‐associated bacteria. Approximately, 75% of the bacterial taxa identified in biopsies were reflected in fecal samples. NGS failed to detect low‐abundance CRC‐associated taxa in fecal samples, whereas qPCR exhibited high sensitivity and specificity in identifying all targeted taxa. Comparison of fecal microbial composition between the different patient groups showed enrichment of *Fusobacterium nucleatum*, *Parvimonas micra*, and *Gemella morbillorum* in cancer patients. Our findings suggest that low‐abundance mucosa‐associated bacteria can be detected in fecal samples using sensitive qPCR assays.

## Graphical abstract

Colorectal cancer is influenced by various factors, including gut bacteria. This study has investigated gut bacteria in biopsies and fecal samples from cancer patients, patients with adenomatous polyps, and healthy individuals. Our results revealed an enrichment of oral‐originated bacteria in cancer patients compared to the other groups and demonstrated that these bacteria can be detected in feces.

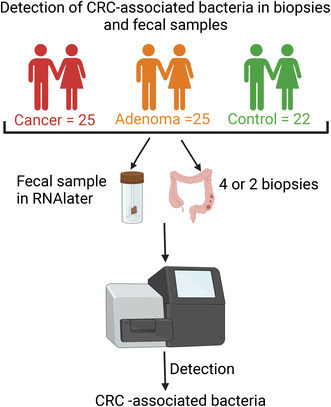

**Abbreviations**
ASV: amplicon sequence variant
BLAST: basic local alignment search tool
CCUG: Culture Collection University of Gothenburg
CRC: colorectal cancer
Fad A: *Fusobacterium* adhesin A
gFOBT: guaiac‐based fecal occult blood test
iFOBT: human hemoglobin immunochemical‐based fecal occult blood test
LOD: limit of detection
MIQE: minimum information for publication of quantitative real‐time PCR experiments
NCBI: National Center for Biotechnology Information
NGS: next‐generation sequencing
PCoA: principal coordinate analysis
QIIME2: quantitative insights into microbial ecology
qPCR: quantitative polymerase chain reaction
rRNA: ribosomal RNA

## 1 Introduction

Colorectal cancer (CRC) is the third most common cancer worldwide with over 1.9 million new CRC cases and 930 000 deaths in 2020 [1]. Randomized clinical trials have shown that early diagnosis leads to improved outcomes for CRC patients [2, 3]. Growing incidence of CRC and the importance of early diagnosis have led to population‐wide screening initiatives worldwide. Varieties of methods, such as guaiac‐based fecal occult blood test (gFOBT), human hemoglobin immunochemical‐based fecal occult blood test (iFOBT), sigmoidoscopy, and colonoscopy, are used for screening of CRC [4, 5]. The Norwegian health authorities have implemented a national screening program, using iFOBT for diagnostics of CRC for all 55 years old [6]. Large variation in sensitivities of iFOBT test has been observed depending on the cancer stage and the location of the tumor. The sensitivity for detecting cancers and large adenomas in the proximal colon is lower than detecting in the distal colon. Similarly, the sensitivity for detection of stage I cancer is lower than the sensitivity in the detection of stages II–IV [7, 8, 9, 10]. Thus, European Cancer Screening guidelines recommend a screening interval of maximum of 2 and 3 years for gFOBT and iFOBT, respectively [11]. Given the need for improved non‐invasive screening, finding additional biomarkers for diagnosis of CRC has been in focus in recent years.

It has been known for several years that the gut microbiota is associated with CRC development, and studies have reported enrichment of specific bacteria such as *Fusobacterium nucleatum*, *Parvimonas micra*, *Gemella morbillorum*, and *Peptostreptococcus stomatis* repeatedly in cancer patients [12, 13, 14, 15, 16]. A review by Zhang et al. [17] identified three genera as overlapping in six metagenomic studies across populations: *Porphyromonas*, *Parvimonas*, and *Peptostreptococcus*. Identification of the same bacterial taxa in different studies independent of geography illustrates their association with CRC. Furthermore, detection of these bacteria together leads to hypotheses suggesting that these bacteria may coexist in polymicrobial biofilms [18, 19, 20]. Thus, detecting CRC‐associated bacteria in fecal samples as non‐invasive biomarkers for CRC has been suggested as a screening method. However, the ability of fecal samples to reflect the microbial composition at mucosal biopsies has been debated [21, 22].

Several studies have examined fecal samples to identify the bacteria that distinguish CRC patients from healthy controls and most of them utilized next‐generation sequencing (NGS) of 16S ribosomal RNA amplicons [12, 23, 24, 25, 26]. Fecal samples are complex and heterogeneous, containing a wide range of microbial species at varying abundances. The presence of abundant bacteria can mask the detection of low‐abundance bacteria, making it challenging to detect these by NGS. Additionally, 16S rRNA sequencing is unable to distinguish all bacteria at the species level. Species‐specific quantitative PCR (qPCR) assays have been used to detect low‐abundant CRC‐associated bacteria from fecal samples [14, 15, 27]. However, sensitive qPCR assays have not been established for all bacteria found to be associated with CRC, for example, *Leptotrichia* spp. and *Porphyromonas endodontalis*. There is a paucity of studies that have established and analyzed a large panel of qPCR assays for CRC‐associated bacteria. In addition, only a limited number of studies have had access to both biopsies and stool samples for comparison [28, 29].

In our earlier research, we detected increased levels of distinct microbial taxa within mucosal biopsy samples from colorectal cancer patients by employing 16S rRNA amplicon sequencing. Our analyses revealed that the bacterial genera *Fusobacterium*, *Gemella*, *Parvimonas*, *Granulicatella*, *Leptotrichia*, *Peptostreptococcus*, *Porphyromonas*, *Prevotella*, *Campylobacter*, and *Selenomonas* were significantly more abundant in biopsy specimens obtained from patients with cancer compared to those from individual with adenomatous polyps and healthy controls [30].

Following prior research, the aim of this study was to investigate the detection of the same CRC‐associated bacteria in fecal samples and to assess the suitability of fecal samples as non‐invasive material. 16S rRNA amplicon sequencing and 19 newly established qPCR assays were used to assess the detection of CRC‐associated bacteria in fecal samples.

## 2 Materials and methods

### 2.1 Study population and sample collection

Seventy‐two subjects with scheduled colonoscopy at Akershus University Hospital, Norway, from 2014 to 2017 were included in the study. The inclusion criteria for the study were changes in stool patterns persisting for more than 4 weeks, unexplained bleeding, or findings of polyps by CT colonography. Participants with known inflammatory bowel disease were excluded from the study. Prior to colonoscopy, participants were informed about the study and their rights to withdraw from it. Written informed consent was obtained from all included participants. The participants were grouped into three based on the findings during colonoscopy, cancer patients (*n* = 25), patients with adenomatous polyp over 1 cm (*n* = 25), and individuals with no pathological findings at colonoscopy were defined as healthy controls (*n* = 22) (Table S1a–c). Each participant collected one spoon of fecal sample in 4 mL RNAlater RNA stabilizing buffer (Thermo Fisher Scientific, Waltham, MA, USA) either prior to colonoscopy or at least 1 week after colonoscopy [31]. Fecal samples were homogenized and stored at −80 °C until extraction. Two cancer patients did not provide fecal samples, leaving the cancer group with *n* = 23. Colonic mucosal biopsies (sized 2–3 mm) were collected from tumor, adjacent non‐neoplastic tissue, ascending colon, and colon sigmoideum from cancer patients and patients with adenomatous polyps. In control individuals, biopsies were collected from ascending colon and colon sigmoideum. Biopsies were stored in Allprotect Tissue Reagent (Qiagen, Hilden, Germany) (for details, see Senthakumaran et al. [30]).

### 2.2 Extraction of DNA from fecal samples

DNA was extracted from all RNAlater‐preserved fecal samples using PSP® Spin Stool DNA Kit (Stratec Molecular Gmbh, Berlin, Germany) as described elsewhere [32]. Briefly, 200 mg of stool sample was extracted with the bacterial DNA enrichment protocol according to the manufacturers' recommendations, except the mechanical lysis with FastPrep‐24 (MP Biomedicals, Irvine, CA, USA) at 6 m·s^−1^ for 3 × 1 min. The DNA was eluted using 100 μL elution buffer and was stored at −20 °C.

### 2.3 16S rRNA amplicon sequencing analysis

In this study, we sequenced 70 fecal samples (cancer = 23, adenomatous polyp = 25, and control = 22) using 16S rRNA V4 region as target. 16S rRNA amplicon sequencing and data analysis were performed as described previously [30]. Briefly, 16S rRNA amplicon sequencing was performed using 16S forward primer (16Sf V4: GTGCCAGCMGCCGCGGTAA) and 16S reverse primers (16Sr V4: GGACTACHVGGGTWTCTAAT) [33]. Pooled amplicon libraries of around 400 bp were purified from 3% agarose gel using QIAquick Extraction Kit (Qiagen). Gel‐purified amplicons were quantified using Kapa Library Quantification Kit (Universal) (Kapa Biosystems Inc., Wilmington, MA, USA). The sequencing was performed on the Illumina MiSeq platform (Illumina Inc., San Diego, CA, USA). FastQ file generation was performed using miseq reporter software (Illumina Inc.). Raw FastQ reads were quality filtered, trimmed, de‐noised, and paired‐end sequences merged, using dada2 [34] and the q2‐dada2 plugin implemented in qiime2 (Quantitative Insights Into Microbial Ecology) version 2021.2.0. In brief, after inspection of the quality of the sequences, 250 bases were kept for both R1 and R2 files. Otherwise, default settings in the q2‐dada2 plugin were used. Taxonomy was assigned to Amplicon Sequence Variants (ASVs) using a pre‐trained Naive Bayes classifier, trained on the Silva V.138 reference sequence database. Analysis of NGS data was performed in r (version 4.1.2) using packages tidyverse (version 1.3.2) and phyloseq (version 1.36.0). ASV counts were converted into relative abundance using the transform_sample_counts function within the phyloseq package.

Data from 16S rRNA V4 region amplicon sequencing of mucosal biopsy samples from the same participants were used to assess the suitability of the fecal samples for detection of CRC‐associated bacteria (for details, see Senthakumaran et al. [30]).

### 2.4 Quantitative PCR analysis

Based on the findings from our previous study [30], we designed and validated 19 qPCR assays for detection of different species within the genera *Fusobacterium*, *Leptotrichia*, *Gemella*, *Campylobacter*, *Granulicatella*, *Porphyromonas*, *Peptostreptococcus*, and *Prevotella* (Table 1). The selection of species for qPCR analysis was mainly based on the data from the previous study of 16S rRNA amplicon sequencing of biopsy samples. Although we could not identify the taxa at species level, further search of the significant ASVs in the National Center for Biotechnology Information (NCBI) Nucleotide database revealed higher degree of similarity with certain species (*P. endodontalis*, *Prevotella copri*, *Campylobacter consisus*). Genera *Gemella* and *Granulicatella* cannot be characterized at species level using 16S rRNA, we therefore designed assays for the most well‐known species [35, 36]. Our previous study of characterization of *Fusobacterium* species and subspecies revealed presence of four subspecies of *F. nucleatum*, and the species *Fusobacterium gonidiaformans*, *Fusobacterium pseudoperiodonticum*, and *Fusobacterium necrophorum* in biopsy samples. Therefore, we designed three qPCR assays for detection of these taxa. Additional species of above‐mentioned genera were included based on previous literature (*P. micra*, *P. stomatis*, *Porphyromonas gingivalis*, and *Porphyromonas asaccharolytica*) [12, 14, 15, 37, 38]. *Leptotrichia* has been linked to CRC, yet no studies have investigated this taxon at the species level. We designed five species‐specific qPCR assays for different *Leptotrichia* spp. Additionally, two qPCR assays were selected from published literature and adapted to our protocol, for detection of *P. micra* and measuring the total bacterial load by targeting the 16S rRNA [39].

**Table 1:** Primer/probe sequences and reaction conditions of the assays used in this study.

Bacteria	Target	Primer and probe sequence	Reference	Final concentration (nm)	Annealing temperature (°C)	Limit of detection	PCR efficiency %
*Fusobacterium nucleatum* ssp *animalis* *Fusobacterium nucleatum* ssp *nucleatum* *Fusobacterium nucleatum* ssp *vincentii* *Fusobacterium nucleatum* ssp *polymorphum*	nusG	Fwd: GCTTGAAATGGAAGCWACAAGAG	This study	400	56	10 fg·μL^−1^	97
		Rev: CCAACTCCTACAAAYCCAGTAAC		400			
		Fam: AGTAGATCCTCGTGTATGGTATGAAGT		400			
*Fusobacterium gonidiaformans* *Fusobacterium necrophorum*	nusG	Fwd: CAGACCCTACTCCAACAAATCC	This study	400	58	1 pg·μL^−1^	88
		Rev: GACGGCAGTACGTGAAGAAA		400			
		YAK: TCGGACTACATACCAAGCATCTGAATCT		300			
*Fusobacterium periodonticum* *Fusobacterium pseudoperiodonticum*	nusG	Fwd: CTATAAAGTAGATCCTGATGTGTGGT	This study	400	58	100 fg·μL^−1^	98
		Rev: TCGTCATCTTCCATAGGAATTGG		400			
		CY3: CGGAGTTACTGGTTTCGTAGGAGTAGGT		300			
*Gemella morbillorum*	rpoB	Fwd: GCTGCTAAACAGTTGGGTATTC	This study	400	58	100 fg·μL^−1^	107
		Rev: GTCTTGGCATCTTTAGCCATTC		400			
		YAK: TGCTTCTGCGATAGTTGCCCAAACATC		300			
*Gemella haemolysans* *Gemella parahaemolysans* *Gemella taiwanensis*	rpoB	Fwd: CACCGAAAGGATTAACTGAACAA	This study	400	60	100 fg·μL^−1^	97
		Rev: TAACATCAGCAACTACTCCATC		400			
		Fam: TCATTACGTGTACCTCACGGTGC		300			
*Gemella sanguinis*	rpoB	Fwd: CGGAGCTAAATCTAAAGAGGTACG	This study	400	58	100 fg·μL^−1^	98
		Rev: TAGCAAGTTCTGCTCCATCTTC		400			
		YAK: ATCCGCAACTACTCCATCAGCACC		300			
*Granulicatella adiacens* *Granulicatella elegans*	rpoB	For: AGAAATCACGACGCAAATTA	This study	400	54	100 fg·μL^−1^	97
		Rev: CCAAAGAAKACTTCTTGGTCTTT		400			
		FAM: AATGCGTTTAGTGAAYAAAGAAACTGG		300			
*Granulicatella adiacens*	rpoB	For: TATTCACCAGCTGACCCAGAA	This study	400	54	100 fg·μL^−1^	97
		Rev: CGATAATGTCGCTTGCAGTCA		400			
		Fam: CGGAAACGTGACGGAAGACGTGAAAC		400			
*Leptotrichia hofstadii*/*Leptotrichia buccalis*	16S rRNA	Fwd: AGGGATAACAGACGGAAACGAC	This study	400	56	1 pg·μL^−1^	91
		Rev: AGCTAATAGGACGCAARGCTCTC		400			
		FAM: CACGCATGTGCCCGGCAATGA		400			
*Leptotrichia trevisani*	16S rRNA	Fwd AGGGATAACAGACGGAAACGAC	This study	400	56	100 fg·μL^−1^	102
		Rev: AGCTAATAGGACGCAARGCTCTC		400			
		FAM: TGTTGTCAGGCTGACGCATGTCAGGC		400			
*Leptotrichia wadei*	16S rRNA	Fwd: AGGGATAACAGACGGAAACGAC	This study	400	56	1 pg·μL^−1^	95
		Rev: AGCTAATAGGACGCAARGCTCTC		400			
		YAK: GCGGACTCATGTCCAGCCTGATGAAA		400			
*Leptotrichia shahii*	16S rRNA	Fwd: AGGGATAACAGACGGAAACGAC	This study	400	56	100 fg·μL^−1^	102
		Rev: AGCTAATAGGACGCAARGCTCTC		400			
		YAK: ATGTGCCTGGCGATGAAAGGAGACG		400			
*Leptotrichia goodfellowii*	16S rRNA	Fwd: TGGGATAACAGAGGGAAACTTC	This study	400	56	10 fg·μL^−1^	98
		Rev: GCTAATAGGACGCAAAGCTCTC		400			
		FAM: TGATTGCATGAGAGATTAATGAAAAGAGAT		400			
*Peptostreptococcus stomatis*	16S rRNA	Fwd: CGAGGGTTTGCTCAGTATTG ^a^	This study	500	62	1 pg·μL^−1^	87
		Rev: TTATCCATGTGTATAGGGCAG		500			
		Fam: CTAGAATGTTCAATTCTGAGCAAAACC		300			
*Porphyromonas endodontalis*	rpoB	Fwd: GTAAGAAGAAGGTGCCCGTAG	This study	500	56	1 pg·μL^−1^	94
		Rev: CTACAACCTCTCCAATCCCAAA		500			
		Fam: AGCTCCGACCGTGATATCTGGAGT		400			
*Porphyromonas gingivalis*	rpoB	Fwd: GTATACAGGCAGCTCCGTAATG	This study	400	54	1 pg·μL^−1^	98
		Rev: CGCCCAAGTCGTATCGTTTAT		400			
		Fam: TGCAGACGATGCAAGTGCTAGAGA		400			
*Porphyromonas asaccharolytica*	rpoB	Fwd: GGTGCGAGACGTACATTACA	This study	400	56	100 fg·μL^−1^	100
		Rev: GCATAGCGAGGAGATAAGTCC		400			
		Fam: CGAGACGCCTGAGGGTCCAAACATT		400			
*Prevotella copri*	rpoB	Fwd: AGAAGTTGTTCTCTCGTGCTATC	This study	400	56	100 fg·μL^−1^	102
		Rev: CCTTTGCCTCATACTCCTCATC		400			
		Fam: ACCCGTGAGTCAAAGAAGCAGGAT		400			
*Campylobacter concisus*	rpoB	Fwd: TGAGATGGCAAAGATAGATAGCG	This study	400	60	1 pg·μL^−1^	90
		Rev: CATCAACCAAGTGGTGAAGTTT		400			
		Fam: CTATATGACGGACGCACAGGCTC		300			
*Parvimonas micra*	rpoB	Fwd: AAGAATGGAGAGAGTTGTTAGAGAAAGAA	Yu J et al. [18]	500	60	100 fg·μL^−1^	92
		Rev: TTGTGATAATTGTGAAGAACCGAAGA		500			
		Fam: AACTCAAGATCCAGACCTTGCTACGCCTCA		250			
Total bacterial load	16S rRNA	Fwd: AATAAATCATAAACTCCTACGGGAGGCAGCAGT	Brukner et al. [39]	500	50	1 pg·μL^−1^	88
		Rev: AATAAATCATAACCTAGCTATTACCGCGGCTGCT		500			
		Fam: CGGCTAACTMCGTGCCAG		500			

^a^ Modified from Purcell et al. [38].

### 2.5 Design of primers and probes

The target gene for each species included in this study is listed in Table 1. Three or more reference sequences for each target gene were obtained from NCBI Reference Sequence Database and aligned using clustalw Multiple Alignment tool in BioEdit Sequence Alignment Editor to infer the homology. The reference sequences used to design the primers and the TaqMan probes are listed in Table S2. IDT PrimerQuest Tool (Integrated DNA Technologies, Leuven, Belgium) with default design parameters for minimum and maximum amplicon size, melting temperatures, and GC content was used for assay design. The primers and the TaqMan probes were checked for secondary structures, melting temperature, and GC content using OligoAnalyzer (Integrated DNA Technologies), and checked by nucleotide blast (Basic Local Alignment Search Tool) offered by NCBI using database nucleotide collection (nr/nt) for homology with unrelated species [40]. The primers and the TaqMan probes were synthesized by TiB Molbiol (Berlin, Germany) and Integrated DNA Technologies and are listed in Table 1.

### 2.6 Optimization of real‐time qPCR

Optimization of annealing temperature was performed using a temperature gradient from 50 °C to 64 °C during the annealing stage. Optimal annealing temperature for the assays was selected based on lowest Ct values and higher fluorescence intensity for positive controls and no amplification curve for negative controls. Varying concentrations of 100, 200, 300, 400, and 500 nm for forward and reverse primers and 200, 300, and 400 nm for probe were tested to determine the optimal concentration based on the same criteria. To establish PCR efficiencies and determine the limit of detection (LOD) of the qPCR assays, DNA from pure bacterial suspensions was serially diluted (10‐fold) from 100 ng·μL^−1^ to 10 fg·μL^−1^ and was run in duplicates. The two lowest concentrations with amplification were run in 10 reactions each to determine the LOD. LOD, annealing temperature, and the final concentration of the primers and TaqMan probes are listed in Table 1. PCR amplification reactions were carried out on QuantStudio5 Real‐Time PCR systems (Thermo Fisher Scientific) using Brilliant III Ultra‐Fast QPCR Master Mix (Agilent, Santa Clara, CA, USA) in 20 μL reactions.

All the optimization reactions were performed using 200 pg DNA from pure cultures of species of interest. DNA was extracted from cultures using PrepMan Ultra Sample Preparation Reagent (Thermo Fisher Scientific). A panel of 50 different bacterial strains was established to evaluate the analytical specificity of the assays. The bacterial strains were mainly obtained from Culture Collection University of Gothenburg (CCUG), and some were clinical isolates collected from Akershus University Hospital (Table S3).

### 2.7 qPCR amplification of bacterial DNA from patient fecal samples

Fecal DNA from all participants was analyzed by the 21 qPCR assays (Table 1). The total bacterial load was measured using 16S rRNA in each sample [39]. We diluted every sample 1 : 2 with PCR grade water to eliminate any possible PCR inhibition. All PCR amplifications were performed on quantstudio5 (Thermo Fisher Scientific) using 20 μL of total reaction volume containing 10 μL of Brilliant III Ultra‐Fast QPCR Master Mix (Agilent) and 2 μL of diluted template DNA. Negative extraction control and multiple positive controls were included in every experiment.

### 2.8 Statistical analysis

Alpha diversity was assessed using Shannon and inverse Simpson indices. Distance matrix was calculated based on Bray–Curtis dissimilarity and UniFrac distance using function adonis2 with 999 permutations (package vegan version 2.6.4). Principal Coordinate Analysis (PCoA) and Permutational analysis of variance (PERMANOVA) were used to visualize the data and compare Bray–Curtis distances with respect to sample type or patient groups using the adonis2 function within the package vegan, version 2.6.4. ASVs with more than 10 reads were retained for further analysis of differential abundance. The phyloseq_to_deseq2 function within the package deseq2 (version 1.32.0) was used to identify differentially abundant taxa between sample types and differentially abundant fecal microbiota between patient groups. Spearman rank correlation was used to assess the relationship in microbial composition between fecal samples and biopsy samples using graphpad prism 9 (GraphPad Software, San Diego, CA, USA).

Data analysis of qPCR results was carried out using quantstudio Design and Analysis Software and the ΔCt method (ΔCt = Ct_target_ − Ct_16SrRNA_). Relative abundance of each target was calculated by 2^−ΔCt^ [41]. All statistical analyses were performed by ibm spss statistics v28 (IBM [International Business Machines], Armonk, NY, USA). The test of normality was performed using the Shapiro–Wilk test in SPSS. The differences between the groups were assessed using the non‐parametric Kruskal–Wallis test. *P*‐value < 0.05 was considered significant for all statistical analyses.

### 2.9 Ethics approval

This study was approved by the regional committee for medical and health‐related research ethics (REK 2012/1944) and the data protection manager at Akershus University Hospital. All experiments were performed in accordance with and following the Declaration of Helsinki Principles. All methods were performed in accordance with the relevant guidelines and regulations.

## 3 Results

### 3.1 Sequence analysis of fecal samples

Sequencing the 16S rRNA gene V4 region of fecal samples collected from 23 cancer patients, 25 patients with adenomatous polyps, and 22 healthy controls provided 2 941 124 raw sequencing reads, representing 2487 taxa. This encompassed 13 phyla, 81 families, and 231 genera. Positive controls had on average 32 766 reads. Negative extraction controls gave < 10 reads.

### 3.2 Comparison of microbial profiles between paired biopsy and fecal sample

**Fig. 1:**
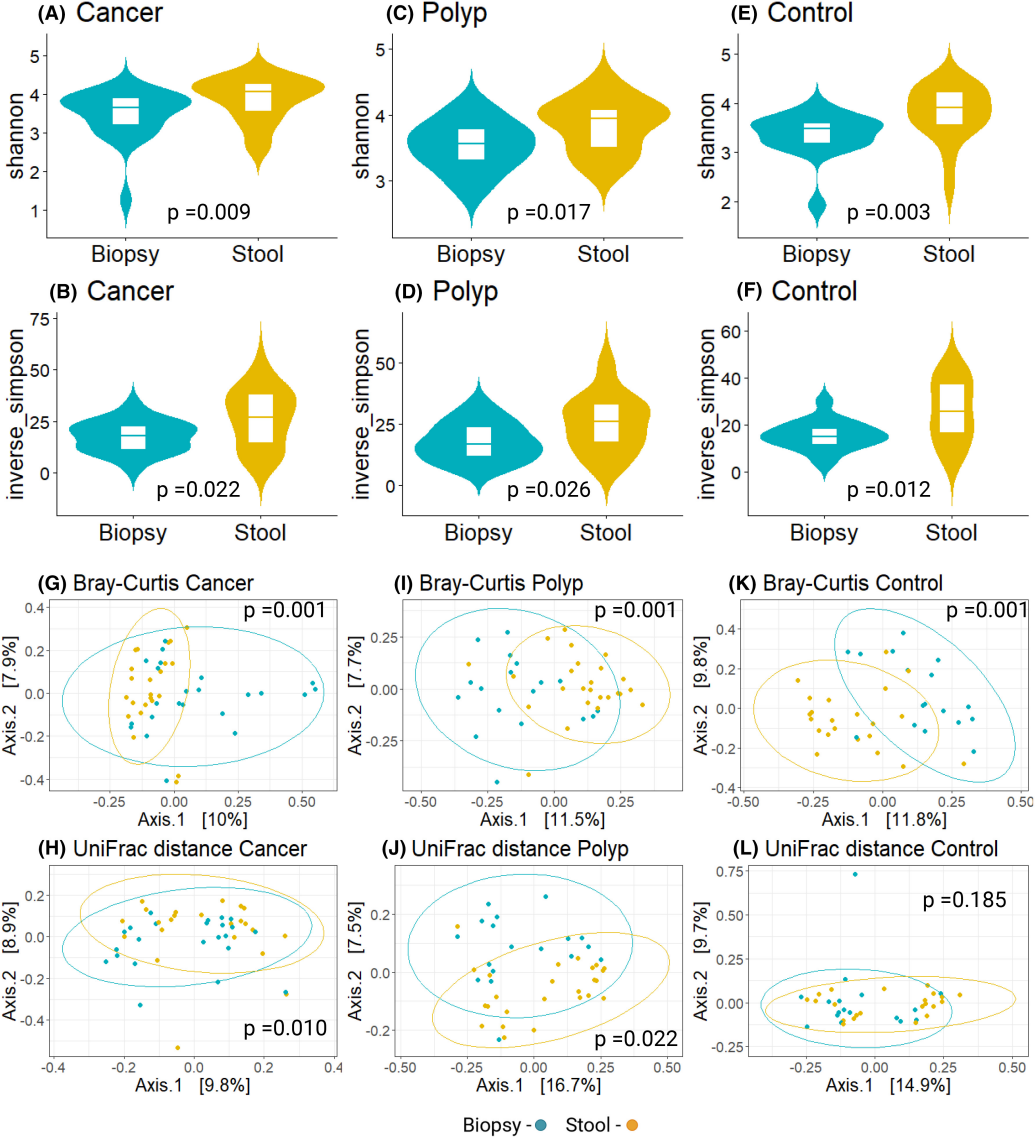
Comparison of microbial profiles between biopsy and fecal samples. (A–F) Violin plots with box plots of Shannon and inverse Simpson alpha diversity indexes in biopsy and fecal samples (Stool) from cancer group, adenomatous polyp (polyp) group, and healthy controls. Kruskal–Wallis test revealed significant differences in diversity indexes between sample types, in all three groups. (G–L) Principal Coordinate Analysis (PCoA) plots based on Bray–Curtis distance matrix and weighted UniFrac distance for biopsies and fecal samples. Permutational analysis of variance (PERMANOVA) showed significant differences in microbial composition between biopsies and fecal samples in all patient groups based on Bray–Curtis dissimilarity. Weighted UniFrac index showed significant differences in cancer group and adenomatous polyp group, but not in healthy controls.

To compare microbial profiles between different sample types, we selected one biopsy from each participant, namely tumor biopsy from cancer patients, polyp biopsy from adenomatous polyp patients, and biopsy from colon sigmoideum from healthy controls. Alpha diversity for fecal microbiota was significantly higher than for mucosal microbiota in all three patient groups, with higher inverse Simpson index and species evenness (Shannon index) (Fig. 1A–F).

### 3.3 Beta diversity indexes reveal significant differences between fecal and mucosal microbiota composition

**Fig. 2:**
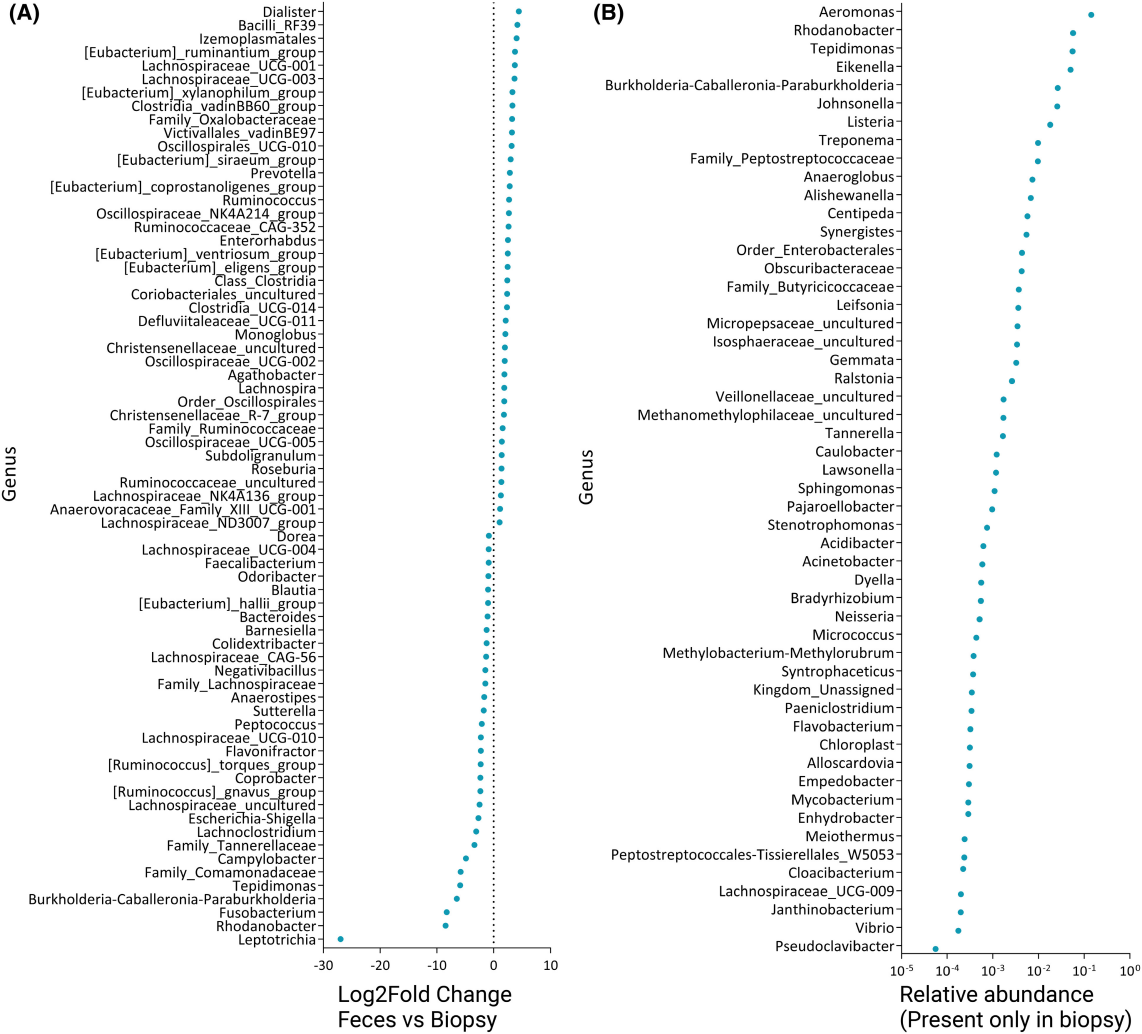
Microbial composition in biopsy and fecal samples. (A) Significantly differentially abundant genera between biopsy and fecal samples. Negative log_2_ fold change means less abundance in fecal samples. The colorectal cancer‐associated genera *Fusobacterium*, *Leptotrichia*, and *Campylobacter* are significantly less abundant in fecal samples. (B) Relative abundance of genera that were only present in biopsy (data from 16S ribosomal RNA (rRNA) amplicon sequencing).

Bray–Curtis and Weighted UniFrac indices were calculated to assess the dissimilarity and phylogenetic diversity, respectively, among the microbial composition in biopsy and fecal samples. Bray–Curtis indices revealed significant dissimilarities between the sample types in all three groups (*P* = 0.001). The weighted UniFrac indices showed significant differences between the sample types in cancer group (*P* = 0.010) and adenomatous polyp group (*P* = 0.022) but not for healthy controls (*P* = 0.185) (Fig. 1G–L). Differential analysis was performed to assess which taxa were driving the differences between sample types. The analysis identified 3, 36, and 70 taxa that were significantly different between mucosal biopsies and fecal samples at phylum, family, and genus levels, respectively. For the bacterial genera focused in the present study, *Fusobacterium*, *Leptotrichia*, and *Campylobacter* were significantly less abundant in fecal samples compared to biopsies (Fig. 2A). In addition, 28 other genera were significantly less abundant in fecal samples compared to biopsies.

### 3.4 Strong correlation in microbial composition between sample types

**Fig. 3:**
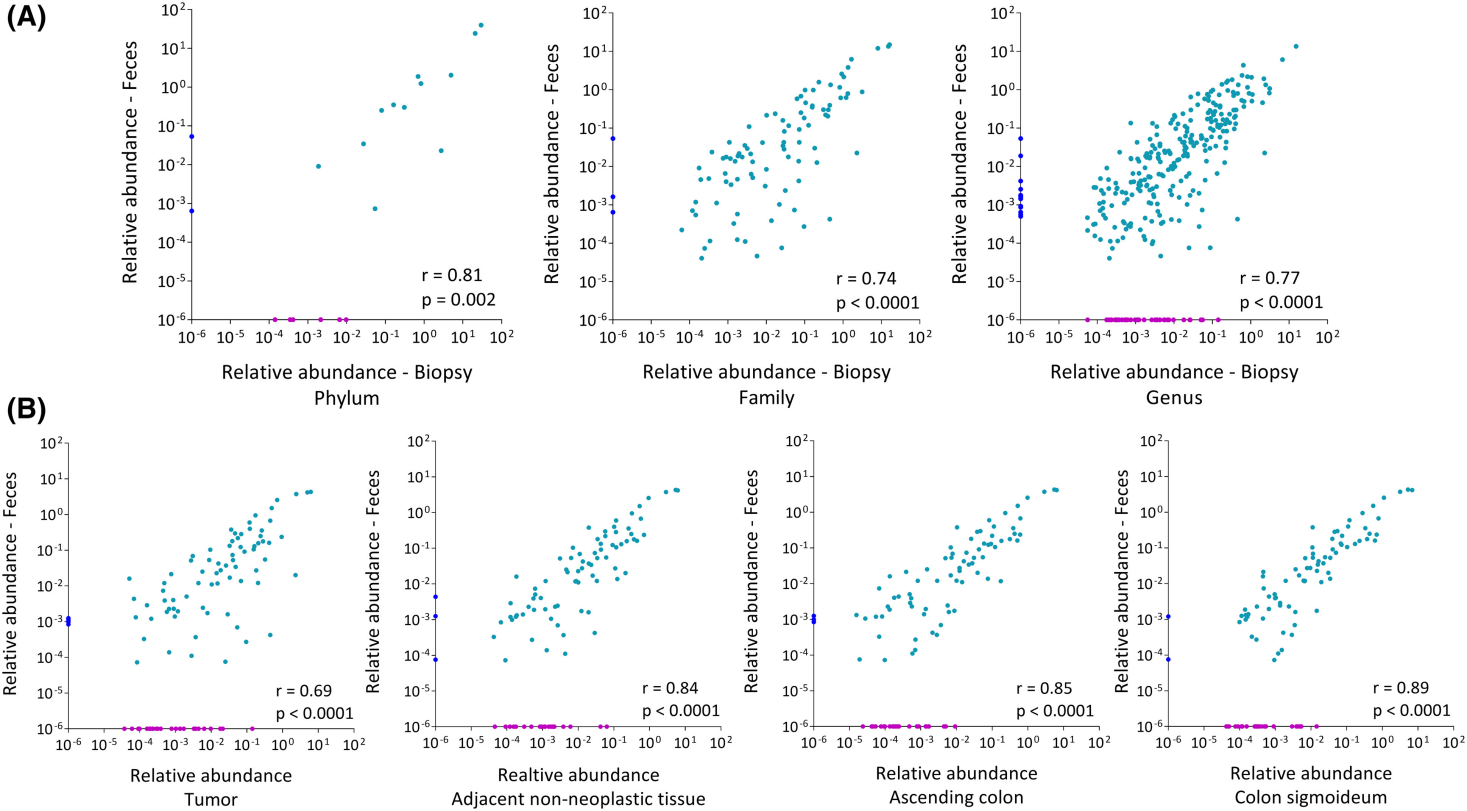
Correlation of relative abundance (obtained from 16S ribosomal RNA (rRNA) amplicon sequencing data) of taxa between sample types. (A) Correlation of relative abundance of identified taxa at phylum, family, and genus levels between paired biopsies and fecal samples from cancer patients, patients with adenomatous polyps and control patients. One biopsy from each participant, namely tumor biopsy from cancer patients, polyp biopsy from adenomatous polyp patients, and biopsy from colon sigmoideum from healthy controls was selected for this analysis. Spearman rank correlation indicates a strong correlation between sample types at all three levels. (B) Spearman correlation of taxa at family level between fecal samples and each biopsy site in cancer patients. Microbial composition at colon sigmoideum showed more similarity to fecal samples (*r* = 0.89). Meanwhile, microbiota at tumor site is less correlated with fecal samples (*r* = 0.69). Taxa with < 10 reads were discarded in both datasets. 0.000001 is added to the sum of relative abundance of all taxa. Dark blue points represent taxa that were present only in fecal samples, and purple points represent taxa that were present only in biopsy samples.

A Spearman rank correlation analysis was conducted to examine the relationship between the relative abundance of identified taxa at different taxonomic levels: phylum, family, and genus, for paired fecal samples and mucosal biopsies. In this analysis, a total of 20 phyla, 121 families, and 321 genera were considered. The results of the analysis revealed a strong positive correlation between mucosal biopsies and fecal samples. Specifically, the correlation coefficients were 0.81 at the phylum level, 0.74 at the family level, and 0.77 at the genus level. This indicates a significant similarity in the microbial composition between these sample types at various taxonomic levels (Fig. 3A). At phylum, family, and genus levels, 60%, 75%, and 79% of taxa were shared among the samples types, respectively. Taxa that were not shared between biopsies and fecal samples were mainly (75%) present only in biopsies. A number of these taxa have been reported as contaminating species from DNA extraction kits and other reagents [42] (Fig. 2B). The correlation between microbial composition at each biopsy site and paired fecal sample from cancer patients revealed higher correlation between colon sigmoideum and fecal samples (*r* = 0.89, *P* < 0.0001). The microbiota composition at tumor site showed lower correlation (*r* = 0.69, *P* < 0.0001) with fecal samples compared to other sites (Fig. 3B).

### 3.5 Comparison of fecal microbial profiles between cancer patients, patients with adenomatous polyp and healthy controls

Kruskal–Wallis rank sum test revealed no significant differences in Shannon and Inverse Simpson diversity indices between the patient groups (*P* = 0.72 and *P* = 0.83, respectively) (Fig. S1). Comparison of fecal bacterial composition from the three patient groups using PCoA based on Bray–Curtis index and weighted UniFrac distance showed no significant differences. When considered together, the first two principal components explained 15.6% and 22.2% of the variation between the groups, respectively. However, these percentages were not significant (*P*‐value = 0.95 and 0.78, respectively) (Fig. S2).

**Fig. 4:**
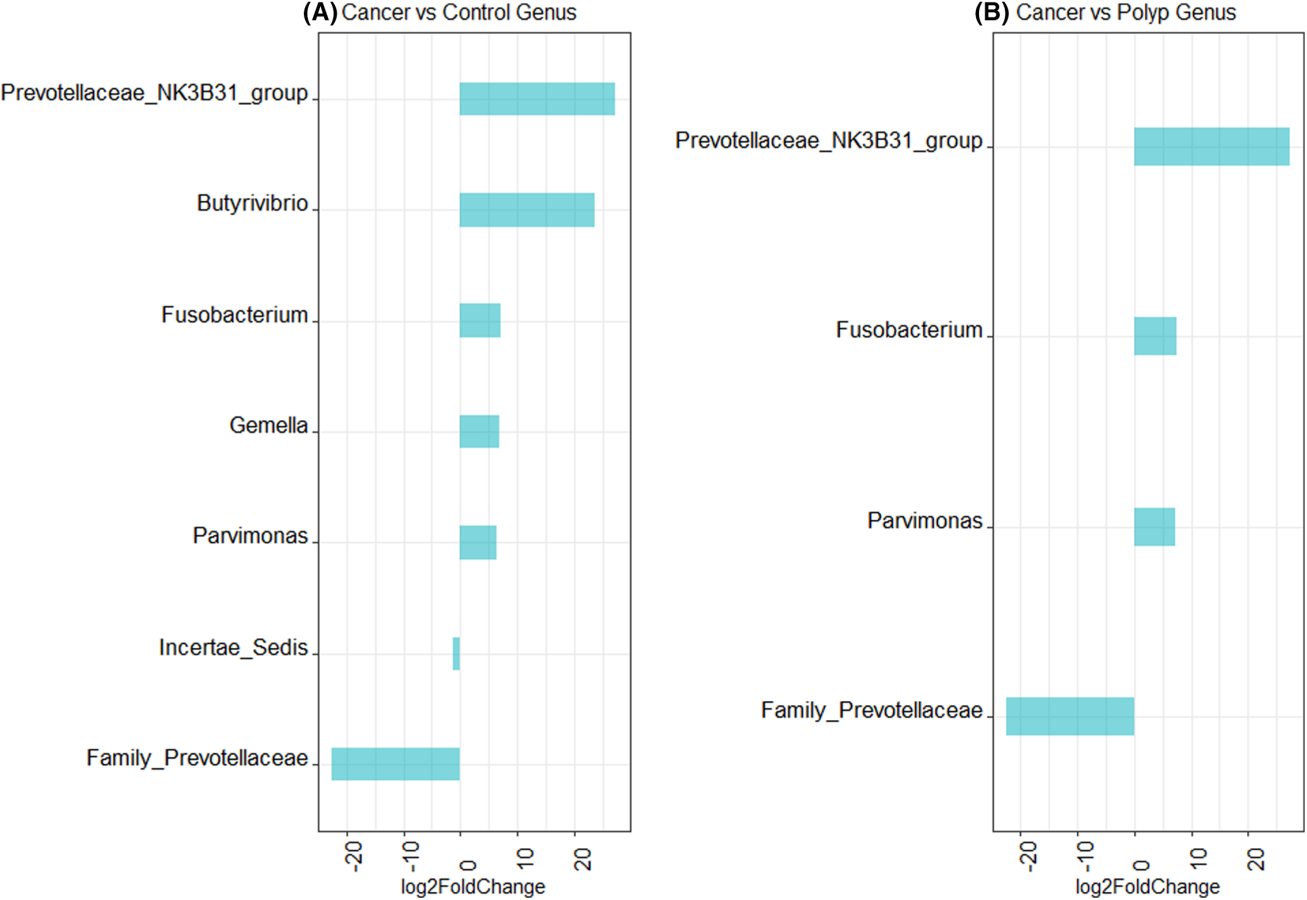
Genera that were enriched and depleted between the cancer group and the adenomatous polyp group or the healthy controls (*P* < 0.05). Data obtained from 16S ribosomal RNA amplicon sequencing. (A) Cancer group vs healthy controls at genus level. (B) Cancer group vs adenomatous polyp group at genus level. Abundance of *Fusobacterium*, *Parvimonas*, and *Prevotellaceae*_NK3B31 were higher in cancer group compared to adenomatous polyp group and healthy controls.

Differential abundance analysis revealed significant differences of taxa at the genus level (Fig. 4). At the genus level, similar to the results obtained from the biopsies, *Fusobacterium*, *Parvimonas*, and *Prevotellaceae*_NK3B31 were enriched in fecal samples from the cancer patients compared to the adenomatous polyp group and the healthy controls. *Gemella* was enriched in the cancer group compared to the healthy controls but not significantly different between the cancer group and the adenomatous polyp group (Fig. 4). In contrast to findings in the biopsy samples, no significant differences were found in the abundances of *Leptotrichia*, *Granulicatella*, *Peptostreptococcus*, *Porphyromonas*, *Campylobacter* or *Selenomonas* between the groups. All significantly abundant taxa are presented in Fig. 4.

### 3.6 Optimization of qPCR assays

Optimal annealing temperature for each assay is listed in Table 1. Analytical specificities of the assays were determined with a panel of 50 various gut bacteria and resulted in 100% specificity for 12 of 20 qPCR assays (Table S3). The *Gemella sanguinis* assay showed cross‐reactions with *Gemella haemolysans* and *Gemella bergeri* with Ct values over 35. Nonspecific reactions were observed in *G. haemolysans* assay beyond 35 cycles of PCR. Therefore, Ct values < 35 were considered as positive for *G. haemolysans* and *G. sanguinis* assays in this study. The *Leptotrichia hofstadii* and *Leptotrichia shahii* assays cross‐reacted with each other. PCR efficiencies determined by standard curves varied between 88% and 107%. The limit of detection and the PCR efficiency are listed in Table 1.

### 3.7 Detection of CRC‐associated bacteria in fecal samples by qPCR

**Fig. 5:**
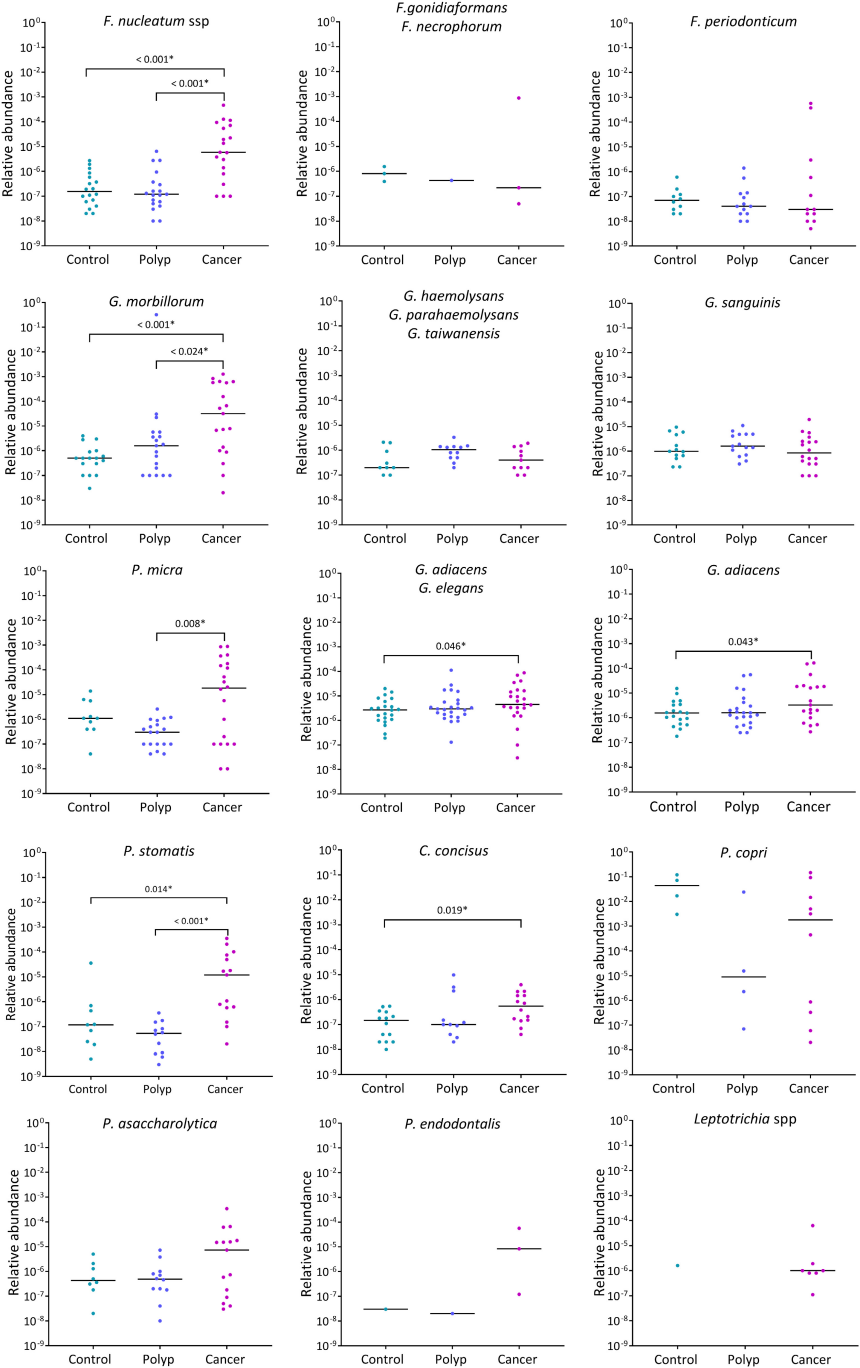
The figure illustrates the presence of colorectal cancer‐associated bacteria in healthy controls, patients with adenomatous polyp and cancer patients. Abundance is plotted in relative abundance (2^−ΔCt^). *Fusobacterium nucleatum* ssp, *Gemella morbillorum*, and *Peptostreptococcus stomatis* are over‐represented in cancer patients compared to patients with adenomatous polyp and healthy controls (Kruskal–Wallis Test). *Granulicatella adiacens* and *Campylobacter concisus* are over‐represented in cancer compared to healthy controls. Meanwhile, *Parvimonas micra* is over‐represented in cancer compared to adenomatous polyp. Each point represents one sample. *Significantly enriched in cancer.

Twenty qPCR assays were employed to detect the presence of selected bacterial species in 70 fecal samples from CRC patients, patients with adenomatous polyps and healthy controls, with one additional qPCR assay for total bacterial load measurement. Distribution of relative abundance of each bacterial species between the three groups is depicted in Fig. 5. Comparison of relative abundance of each bacterium between the groups was performed using the Kruskal–Wallis test. The results showed significant differences in the abundance of *F. nucleatum* ssp. (*P* < 0.001), *G. morbillorum* (*P* < 0.001), *P. stomatis* (*P* = 0.014), *Granulicatella adiacens* (*P* = 0.043), and *Campylobacter concisus* (*P* = 0.019) when comparing the cancer group with the healthy controls. Furthermore, we found significant differences in *F. nucleatum* ssp. (*P* < 0.001), *G. morbillorum* (*P* = 0.024), *P. micra* (*P* = 0.008), and *P. stomatis* (*P* < 0.001) when comparing the cancer group with the adenomatous polyp group. No significant differences were observed in relative abundance of other bacterial species between the groups. Specifically, *G. haemolysans*, *G. sanguinis*, and *P*. *copri* were detected in almost equal quantities in all three groups. All samples tested positive with *G. adiacens*/*Granulicatella elegans* assay. However, when using *G. adiacens*‐specific assay, we found that only 86%, 92%, and 82% of the samples from healthy controls, adenomatous polyp group and cancer group were positive for *G. adiacens*, respectively. *Leptotrichia* spp. were detected in seven cancer patients and in one healthy control, while *F. gonidiaformans* was detected in three cancer patients, one adenomatous polyp patient, and three healthy controls. However, it was enriched in only one cancer patient.

### 3.8 Methodological differences in detection of low‐abundant bacteria

**Fig. 6:**
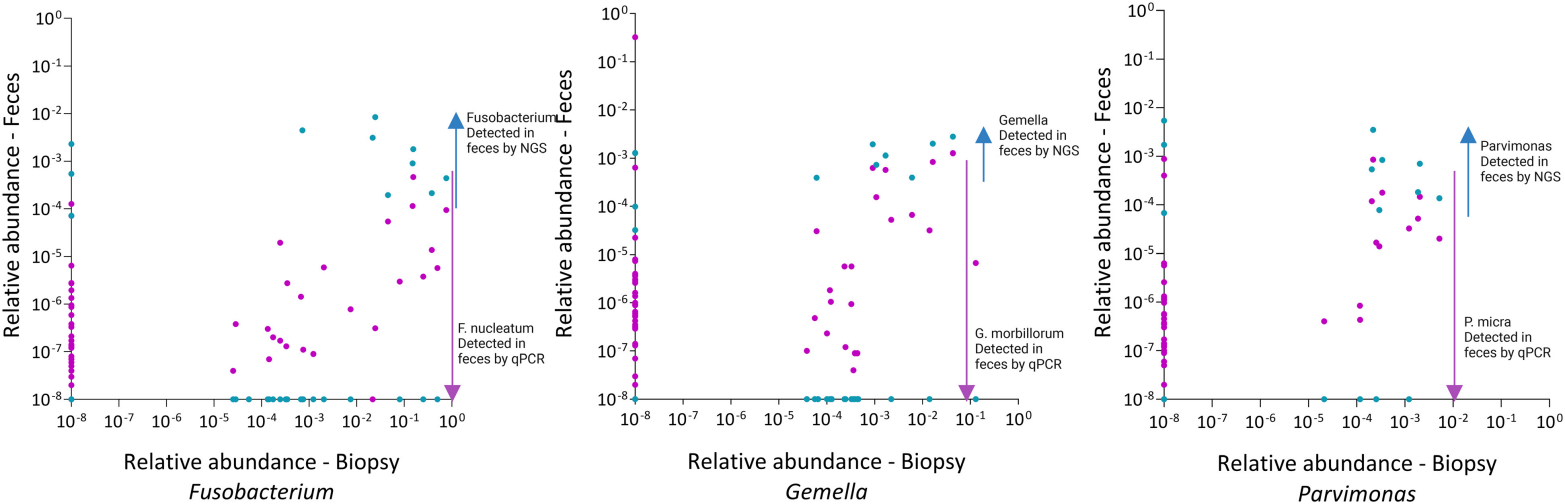
Relationship of relative abundance of *Fusobacterium*, *Gemella*, and *Parvimonas* between fecal samples and biopsies. *X*‐axis – Relative abundance of taxa in biopsy obtained from 16S ribosomal RNA (rRNA) amplicon sequencing. *Y*‐axis – Relative abundance of taxa in fecal sample. Blue points represent relative abundance achieved from 16S rRNA amplicon sequencing analysis, and purple points represent relative abundance achieved from quantitative PCR (qPCR). Each point represents one participant. (To represent negative results, a small number (1 × 10^−8^) was added uniformly to all data points.)

Results from this study revealed that CRC‐associated bacteria were present in low quantities in fecal samples. Comparison of relative abundance of *Fusobacterium* between biopsy and fecal samples from the same individual clearly illustrated higher relative quantities in biopsies compared to fecal samples. This was also the case for *Parvimonas* and *Gemella*. Furthermore, the results demonstrated higher sensitivity of qPCR assays compared to NGS (Fig. 6). If present at sufficient quantities (relative abundance ≥ 10e‐4) in fecal samples, *Fusobacterium* was detected by NGS. However, if present at low quantities (relative abundance ≤ 10e‐4) NGS failed to detect the presence of these bacteria. Conversely, qPCR was able to detect presence of *F. nucleatum* in fecal samples at very low quantities, illustrating the superior sensitivity of qPCR for detection of specific bacteria (Fig. 6).

## 4 Discussion

In studies focusing on gut microbiota, fecal samples are often preferred over biopsies due to their non‐invasive nature and ease of collection. While tumor samples provide information about the microbial composition in a specific region of the gut, which may be relevant for studying CRC‐associated bacteria, fecal samples provide a holistic view of the gut microbiota, capturing the overall composition. Fecal microbial composition is dynamic and influenced by diet [43], stool consistency [44], and oxygen availability [45]. Thus, the ability of fecal samples to represent the microbial composition in the colonic mucosa has been debated. While some studies report similarities between fecal and mucosal bacteria compositions [21, 22], others claim that the mucosal bacteria differ from those in feces [46].

In our previous study, we identified enrichment of bacteria associated with CRC in mucosal tumors from cancer patients. In the present study, fecal samples from the same participants were analyzed to evaluate the representativeness of fecal sample as a non‐invasive sample material for detection of CRC‐associated bacteria. The samples were analyzed using 16S rRNA amplicon sequencing and species‐specific probe‐based qPCR assays. 16S rRNA gene sequencing‐based bacterial composition was compared between biopsy and fecal samples.

The results illustrate that approximately 75% of the bacteria identified in biopsies can be reflected in fecal samples. Diversity analyses demonstrated significant differences in the bacterial composition between fecal samples and biopsies. These differences can be explained by the taxa being differently abundant in the two sample materials and the taxa not present in fecal samples. Certain genera were exclusively detected in biopsy samples and among these, *Acinetobacter*, *Ralstonia*, *Bradyrhizobium*, *Leifsonia*, *Methylobacterium*, *Burkholderiaceae*, *Stenotrophomonas*, *Cloacibacterium*, *Enhydrobacter*, *Flavobacterium*, *Micrococcus*, *Tepidimonas*, and *Sphingomonas* have been identified as contaminants originating from laboratory environment [21, 42, 47, 48]. Mucosal biopsies have varying bacterial biomass, and samples with low bacterial biomass have been shown to be highly susceptible to contamination in 16S rRNA amplicon sequencing [42]. Fecal samples are high bacterial biomass samples and are not likely to be equally affected by contamination. Our data support the literature that the CRC‐associated microbiome is reflected in fecal samples.

Detailed analysis of microbial profiles and sample type in cancer patients showed that the highest correlation was observed between colon sigmoideum biopsy and fecal samples and the lowest correlation was observed between tumor biopsy and fecal samples. The results illustrate that the tumor microenvironment differs from other positions in the colon. Our analyses further showed that the CRC‐associated bacteria were present in low numbers in fecal samples, making them prone to being outcompeted in amplicon‐based NGS with universal primers. This was shown in the present study where many of the CRC‐associated taxa were not identified in fecal samples using NGS. The findings suggest that detecting mucosa‐associated bacteria in fecal samples using 16S rRNA sequencing should be carefully considered. Furthermore, 16S rRNA amplicon sequencing may not always classify the bacteria at species level. To overcome these problems, 19 high sensitive, species‐specific qPCR assays were established and thoroughly validated according to the MIQE guidelines [49]. The results showed that qPCR was able to detect all CRC‐associated species also in fecal samples and illustrate that choice of methodology is highly important.

Several studies have employed qPCR for the detection of CRC‐associated bacteria, particularly *Fusobacterium* [50, 51, 52]. However, not all studies have consistently reported essential parameters such as PCR efficiency and limit of detection. Moreover, many studies have used SYBR‐green‐based PCR assays. We developed TaqMan‐probe‐based qPCR assays to enhance the specificity of the assays. Additionally, efforts were made to standardize annealing temperatures, thereby enabling the potential for multiplexing assays if required. Furthermore, in the absence of existing PCR methods for detection of *Leptotrichia*, we developed five distinct assays to characterize *Leptotrichia* species associated with CRC.

The qPCR results revealed that *F. nucleatum*, *G. morbillorum*, *G. adiacens*, and *P. micra* were detected in over 70% of the samples in cancer patients, patients with adenomatous polyps and healthy controls. However, an enrichment of *F. nucleatum*, *G. morbillorum*, and *P. micra* in the cancer group compared to the other two groups could be seen. The increased abundances of these species, normally present in low numbers in healthy controls, strongly indicate an association with CRC. *F. nucleatum* is extensively studied with regard to its role in CRC development and is believed to promote CRC through various mechanisms. One of the mechanisms is *Fusobacterium* adhesin A (FadA), an adhesive membrane protein, that facilitates adhesion and invasion of the bacterium to the host epithelial cells [53] and selectively stimulates growth of cancerous cells [54, 55]. The present study found *G. morbillorum* to be enriched in the cancer group compared to the adenomatous polyp group and healthy controls. Interestingly, the species‐specific qPCR assays did not show any differences in abundances of *G. haemolysans* or *G. sanguinis* between the groups, indicating no association with CRC. *G. morbillorum*, a Gram‐positive, facultative anaerobic microbe, has been detected in various clinical infections, such as endocarditis or abscesses. However, there is limited research specifically linking this bacterium to CRC [56, 57]. *P. micra* is a Gram‐positive anaerobic microbe found in the oral cavity, gastrointestinal tract, and respiratory tract [58]. An increasing number of studies have reported increased abundance of *P. micra* in CRC patients compared to colorectal adenoma and healthy controls [14, 59]. However, the exact mechanisms by which *P. micra* may contribute to cancer are not fully understood. Studies have recently suggested that *P. micra* may induce epigenetic changes in host epithelial cells through the NF‐kB signaling pathway [60] and may associate with immune responses in CRC [61].

In consistence with other studies, our previous study demonstrated an enrichment of genus *Porphyromonas* in tumors from cancer patients. Similarity search of the significant *Porphyromonas* ASV in NCBI nucleotide database revealed a higher degree of similarity to *P. endodontalis*. We therefore established qPCRs for this species, for *P. gingivalis* associated with CRC, and for *P. asaccharolytica* previously found in sepsis. Although the abundance of *P. asaccharolytica* was not found to be significantly different between the groups, 65% of the cancer patients were positive for *P. asaccharolytica*, while only 36% and 48% of the healthy controls and adenomatous polyp patients were positive, respectively. *P. asaccharolytica* and *P. endodontalis* were detected in all three groups, but none of the samples were positive for *P. gingivalis*. *P. gingivalis*, a well‐studied periodontopathogen and commonly associated with periodontal disease, has been linked to CRC [62]. However, in the study done by Kerdreux et al. [62], only a small fraction (2–5%) of CRC patients tested positive for *P. gingivalis*, indicating a low occurrence rate. It is noteworthy that our study comprised a small number of CRC patients, potentially contributing to the absence of positive samples. Furthermore, our data suggest that several *Porphyromonas* species may have a role in CRC.

The *Granulicatella* assays showed significantly higher abundances in the cancer group compared to the healthy controls. Although statistically significant, the difference is small. The majority of the healthy controls also were positive for *G. adiacens* with relatively high abundance. This observation suggests that *Granulicatella* may be frequently present in stool samples as a part of the normal flora irrespective of disease status, as opposed to *Fusobacterium*, whose abundance was very low in healthy controls. The significantly higher abundance of *G. adiacens* in mucosa and fecal samples in cancer patients could indicate that *Granulicatella* spp. have the potential to integrate into biofilms. Our previous study demonstrated enrichment of *Leptotrichia* spp. in cancerous tumors in few cancer patients. Although *Leptotrichia* spp. could not be detected in fecal samples by NGS, it was detected by qPCR in as many samples as biopsy. *Leptotrichia*, belonging to the family *Fusobacteriaceae*, raises speculation about its potential role in bacterial aggregation, similar to that of *Fusobacterium*.

Results from this study illustrate that 16S rRNA amplicon sequencing provides a comprehensive analysis of the bacterial composition in the gut, including the identification of CRC‐associated bacteria. However, this method struggles to detect low‐abundance bacteria in fecal samples and is time‐consuming and labor intensive. Additionally, shortcoming in species‐level resolution is another limitation of 16S rRNA amplicon sequencing. Certain bacteria associated with colorectal cancer may exhibit species‐level variations that are important to understand their role in CRC development. Thus, reliable high‐resolution identification of the CRC‐associated bacteria is important for diagnosis at an early stage. We acknowledge that the sample size and the collection of fecal samples represent significant limitations in this study. Logistical constraints necessitated the collection of a subset of fecal samples, particularly those from cancer patients, at least 1 week after colonoscopy procedure.

## 5 Conclusion

We have established 19 species‐specific probe‐based qPCR assays and performed thorough validation according to the MIQE guidelines to ensure analytical specificity and sensitivity. Probe‐based qPCR provides rapid, highly species‐specific identification with higher sensitivity compared to NGS. Our data illustrate that the fecal microbiota reflects the mucosal microbiota to a large extent. Low‐abundant CRC‐associated bacteria are not detected in fecal samples using NGS, but qPCR is able to identify all CRC‐associated bacteria with high sensitivity and specificity. Our findings imply that it is feasible to pinpoint reliable microbial biomarkers for screening CRC. Nevertheless, in order to thoroughly evaluate the potential of these assays in screening for CRC, the clinical sensitivity and specificity should be tested in a large study.

## Conflict of interest

The authors declare no conflict of interest.

## Author contributions

HST, VB, and TS conceptualized and designed the study. HST, VB, and TBR supervised the study. TS and TMT performed next‐generation sequencing. SAB was responsible for patient inclusion and collection of biopsy samples. TS, AEFM, and TBR performed bioinformatic analysis. TS designed and performed the qPCR analysis. TS and DJ performed the statistical analysis. TS wrote the first draft of the manuscript. All authors commented on the previous versions of the manuscript and approved the final manuscript.

### Peer review

The peer review history for this article is available at https://www.webofscience.com/api/gateway/wos/peer‐review/10.1002/1878‐0261.13700.

## Supporting information


**Fig. S1.** Alpha diversity.
**Fig. S2.** Beta diversity analysis did not reveal any significant differences in fecal microbial composition between the groups; cancer patients, patients with adenomatous polyps and healthy controls (Bray‐Curtis *P* = 0.95, UniFrac *P* = 0.78).

**Table S1.** Patient information and feature counts from fecal samples.

**Table S2.** Reference sequence.

**Table S3.** Analytical specificity.

## Data Availability

The datasets of the current study are not publicly accessible in compliance with Norwegian legislation regarding general data protection regulation. Data are available from the corresponding author, on reasonable request, pending a material and data transfer agreement and an amendment application to the committee for medical research ethics. The code may be made available upon written request to the corresponding author.

## References

[1] Morgan E , Arnold M , Gini A , Lorenzoni V , Cabasag CJ , Laversanne M , et al. Global burden of colorectal cancer in 2020 and 2040: incidence and mortality estimates from GLOBOCAN. Gut. 2023;72(2):338–344. 36604116 10.1136/gutjnl-2022-327736

[2] Holme Ø , Løberg M , Kalager M , Bretthauer M , Hernán MA , Aas E , et al. Effect of flexible sigmoidoscopy screening on colorectal cancer incidence and mortality: a randomized clinical trial. JAMA. 2014;312(6):606–615. 25117129 10.1001/jama.2014.8266 PMC4495882

[3] Kronborg O , Fenger C , Olsen J , Jørgensen OD , Søndergaard O . Randomised study of screening for colorectal cancer with faecal‐occult‐blood test. Lancet. 1996;348(9040):1467–1471. 8942774 10.1016/S0140-6736(96)03430-7

[4] Ferlizza E , Solmi R , Sgarzi M , Ricciardiello L , Lauriola M . The roadmap of colorectal cancer screening. Cancers (Basel). 2021;13(5):1101. 33806465 10.3390/cancers13051101 PMC7961708

[5] Bretthauer M , Løberg M , Wieszczy P , Kalager M , Emilsson L , Garborg K , et al. Effect of colonoscopy screening on risks of colorectal cancer and related death. N Engl J Med. 2022;387(17):1547–1556. 36214590 10.1056/NEJMoa2208375

[6] Randel KR , Schult AL , Botteri E , Hoff G , Bretthauer M , Ursin G , et al. Colorectal cancer screening with repeated fecal immunochemical test versus sigmoidoscopy: baseline results from a randomized trial. Gastroenterology. 2021;160(4):1085–1096.e5. 33227280 10.1053/j.gastro.2020.11.037

[7] Nakazato M , Yamano H‐O , Matsushita H‐O , Sato K , Fujita K , Yamanaka Y , et al. Immunologic fecal occult blood test for colorectal cancer screening. JMAJ. 2006;49(5/6):203.

[8] Niedermaier T , Balavarca Y , Brenner H . Stage‐specific sensitivity of fecal immunochemical tests for detecting colorectal cancer: systematic review and meta‐analysis. Am J Gastroenterol. 2020;115(1):56–69. 31850933 10.14309/ajg.0000000000000465 PMC6946106

[9] Morikawa T , Kato J , Yamaji Y , Wada R , Mitsushima T , Shiratori Y . A comparison of the immunochemical fecal occult blood test and total colonoscopy in the asymptomatic population. Gastroenterology. 2005;129(2):422–428. 16083699 10.1016/j.gastro.2005.05.056

[10] Lieberman DA , Harford WV , Ahnen DJ , Provenzale D , Sontag SJ , Schnell TG , et al. One‐time screening for colorectal cancer with combined fecal occult‐blood testing and examination of the distal colon. N Engl J Med. 2001;345(8):555–560. 11529208 10.1056/NEJMoa010328

[11] European Commission , Directorate‐General for Health and Consumers , Executive Agency for Health and Consumers , World Health Organization . In: Karsa L , Patnick J , Segnan N , editors. European guidelines for quality assurance in colorectal cancer screening and diagnosis. Luxembourg: Publications Office; 2010.

[12] Zeller G , Tap J , Voigt AY , Sunagawa S , Kultima JR , Costea PI , et al. Potential of fecal microbiota for early‐stage detection of colorectal cancer. Mol Syst Biol. 2014;10(11):766. 25432777 10.15252/msb.20145645 PMC4299606

[13] Kostic AD , Gevers D , Pedamallu CS , Michaud M , Duke F , Earl AM , et al. Genomic analysis identifies association of *Fusobacterium* with colorectal carcinoma. Genome Res. 2012;22(2):292–298. 22009990 10.1101/gr.126573.111 PMC3266036

[14] Löwenmark T , Löfgren‐Burström A , Zingmark C , Eklöf V , Dahlberg M , Wai SN , et al. *Parvimonas micra* as a putative non‐invasive faecal biomarker for colorectal cancer. Sci Rep. 2020;10(1):15250. 32943695 10.1038/s41598-020-72132-1 PMC7499209

[15] Osman MA , Neoh HM , Ab Mutalib NS , Chin SF , Mazlan L , Raja Ali RA , et al. *Parvimonas micra*, *Peptostreptococcus stomatis*, *Fusobacterium nucleatum* and *Akkermansia muciniphila* as a four‐bacteria biomarker panel of colorectal cancer. Sci Rep. 2021;11(1):2925. 33536501 10.1038/s41598-021-82465-0 PMC7859180

[16] Alexander JL , Posma JM , Scott A , Poynter L , Mason SE , Doria ML , et al. Pathobionts in the tumour microbiota predict survival following resection for colorectal cancer. Microbiome. 2023;11(1):100. 37158960 10.1186/s40168-023-01518-w PMC10165813

[17] Zhang H , Wu J , Ji D , Liu Y , Lu S , Lin Z , et al. Microbiome analysis reveals universal diagnostic biomarkers for colorectal cancer across populations and technologies. Front Microbiol. 2022;13:1005201. 36406447 10.3389/fmicb.2022.1005201 PMC9668862

[18] Yu J , Feng Q , Wong SH , Zhang D , Liang QY , Qin Y , et al. Metagenomic analysis of faecal microbiome as a tool towards targeted non‐invasive biomarkers for colorectal cancer. Gut. 2017;66(1):70–78. 26408641 10.1136/gutjnl-2015-309800

[19] Ahn J , Sinha R , Pei Z , Dominianni C , Wu J , Shi J , et al. Human gut microbiome and risk for colorectal cancer. J Natl Cancer Inst. 2013;105(24):1907–1911. 24316595 10.1093/jnci/djt300 PMC3866154

[20] Drewes JL , White JR , Dejea CM , Fathi P , Iyadorai T , Vadivelu J , et al. High‐resolution bacterial 16S rRNA gene profile meta‐analysis and biofilm status reveal common colorectal cancer consortia. NPJ Biofilms Microbiomes. 2017;3:34. 29214046 10.1038/s41522-017-0040-3 PMC5707393

[21] Watt E , Gemmell MR , Berry S , Glaire M , Farquharson F , Louis P , et al. Extending colonic mucosal microbiome analysis‐assessment of colonic lavage as a proxy for endoscopic colonic biopsies. Microbiome. 2016;4(1):61. 27884202 10.1186/s40168-016-0207-9 PMC5123352

[22] Mukhopadhya I , Martin JC , Shaw S , McKinley AJ , Gratz SW , Scott KP . Comparison of microbial signatures between paired faecal and rectal biopsy samples from healthy volunteers using next‐generation sequencing and culturomics. Microbiome. 2022;10(1):171. 36242064 10.1186/s40168-022-01354-4 PMC9563177

[23] Zackular JP , Rogers MAM , Ruffin MT IV , Schloss PD . The human gut microbiome as a screening tool for colorectal cancer. Cancer Prev Res. 2014;7(11):1112–1121. 10.1158/1940-6207.CAPR-14-0129 PMC4221363 25104642

[24] Wu N , Yang X , Zhang R , Li J , Xiao X , Hu Y , et al. Dysbiosis signature of fecal microbiota in colorectal cancer patients. Microb Ecol. 2013;66(2):462–470. 23733170 10.1007/s00248-013-0245-9

[25] Weir TL , Manter DK , Sheflin AM , Barnett BA , Heuberger AL , Ryan EP . Stool microbiome and metabolome differences between colorectal cancer patients and healthy adults. PLoS One. 2013;8(8):e70803. 23940645 10.1371/journal.pone.0070803 PMC3735522

[26] Wang T , Cai G , Qiu Y , Fei N , Zhang M , Pang X , et al. Structural segregation of gut microbiota between colorectal cancer patients and healthy volunteers. ISME J. 2012;6(2):320–329. 21850056 10.1038/ismej.2011.109 PMC3260502

[27] Yao Y , Ni H , Wang X , Xu Q , Zhang J , Jiang L , et al. A new biomarker of fecal bacteria for non‐invasive diagnosis of colorectal cancer. Front Cell Infect Microbiol. 2021;11:744049. 34976850 10.3389/fcimb.2021.744049 PMC8719628

[28] Flemer B , Lynch DB , Brown JM , Jeffery IB , Ryan FJ , Claesson MJ , et al. Tumour‐associated and non‐tumour‐associated microbiota in colorectal cancer. Gut. 2017;66(4):633–643. 26992426 10.1136/gutjnl-2015-309595 PMC5529966

[29] Conde‐Pérez K , Aja‐Macaya P , Buetas E , Trigo‐Tasende N , Nasser‐Ali M , Rumbo‐Feal S , et al. The multispecies microbial cluster of *Fusobacterium*, *Parvimonas*, *Bacteroides* and *Faecalibacterium* as a precision biomarker for colorectal cancer diagnosis. Mol Oncol. 2024;18:1093–1122. 38366793 10.1002/1878-0261.13604 PMC11076999

[30] Senthakumaran T , Moen AEF , Tannæs TM , Endres A , Brackmann SA , Rounge TB , et al. Microbial dynamics with CRC progression: a study of the mucosal microbiota at multiple sites in cancers, adenomatous polyps, and healthy controls. Eur J Clin Microbiol Infect Dis. 2023;42:305–322. 36703031 10.1007/s10096-023-04551-7 PMC9899194

[31] Tunsjø HS , Gundersen G , Rangnes F , Noone JC , Endres A , Bemanian V . Detection of *Fusobacterium nucleatum* in stool and colonic tissues from Norwegian colorectal cancer patients. Eur J Clin Microbiol Infect Dis. 2019;38(7):1367–1376. 31025134 10.1007/s10096-019-03562-7

[32] Noone C , Bemanian V , Hege A , Tunsjø S . DNA‐ekstraksjon: Mengde er ikke synonymt med mangfold Overingeniør ved Genteknologisk seksjon, Avdeling for Tverrfaglig Laboratoriemedisin, Akershus universitets‐ sykehus (Ahus). 2020.

[33] Kozich JJ , Westcott SL , Baxter NT , Highlander SK , Schloss PD . Development of a dual‐index sequencing strategy and curation pipeline for analyzing amplicon sequence data on the MiSeq Illumina sequencing platform. Appl Environ Microbiol. 2013;79(17):5112–5120. 23793624 10.1128/AEM.01043-13 PMC3753973

[34] Callahan BJ , McMurdie PJ , Rosen MJ , Han AW , Johnson AJA , Holmes SP . DADA2: high‐resolution sample inference from Illumina amplicon data. Nat Methods. 2016;13(7):581–583. 27214047 10.1038/nmeth.3869 PMC4927377

[35] García López E , Martín‐Galiano AJ . The versatility of opportunistic infections caused by *Gemella* isolates is supported by the carriage of virulence factors from multiple origins. Front Microbiol. 2020;11:524. 32296407 10.3389/fmicb.2020.00524 PMC7136413

[36] Cargill JS , Scott KS , Gascoyne‐Binzi D , Sandoe JAT . *Granulicatella* infection: diagnosis and management. J Med Microbiol. 2012;61(Pt 6):755–761. 22442291 10.1099/jmm.0.039693-0

[37] Okumura S , Konishi Y , Narukawa M , Sugiura Y , Yoshimoto S , Arai Y , et al. Gut bacteria identified in colorectal cancer patients promote tumourigenesis via butyrate secretion. Nat Commun. 2021;12(1):5674. 34584098 10.1038/s41467-021-25965-x PMC8479117

[38] Purcell RV , Visnovska M , Biggs PJ , Schmeier S , Frizelle FA . Distinct gut microbiome patterns associate with consensus molecular subtypes of colorectal cancer. Sci Rep. 2017;7(1):11590. 28912574 10.1038/s41598-017-11237-6 PMC5599497

[39] Brukner I , Longtin Y , Oughton M , Forgetta V , Dascal A . Assay for estimating total bacterial load: relative qPCR normalisation of bacterial load with associated clinical implications. Diagn Microbiol Infect Dis. 2015;83(1):1–6. 26008123 10.1016/j.diagmicrobio.2015.04.005

[40] Altschul SF , Madden TL , Schäffer AA , Zhang J , Zhang Z , Miller W , et al. Gapped BLAST and PSI‐BLAST: a new generation of protein database search programs. Nucleic Acids Res. 1997;25(17):3389–3402. 9254694 10.1093/nar/25.17.3389 PMC146917

[41] Schmittgen TD , Livak KJ . Analyzing real‐time PCR data by the comparative CT method. Nat Protoc. 2008;3(6):1101–1108. 18546601 10.1038/nprot.2008.73

[42] Salter SJ , Cox MJ , Turek EM , Calus ST , Cookson WO , Moffatt MF , et al. Reagent and laboratory contamination can critically impact sequence‐based microbiome analyses. BMC Biol. 2014;12(1):87. 25387460 10.1186/s12915-014-0087-z PMC4228153

[43] Bourdeau‐Julien I , Castonguay‐Paradis S , Rochefort G , Perron J , Lamarche B , Flamand N , et al. The diet rapidly and differentially affects the gut microbiota and host lipid mediators in a healthy population. Microbiome. 2023;11(1):26. 36774515 10.1186/s40168-023-01469-2 PMC9921707

[44] Vandeputte D , Falony G , Vieira‐Silva S , Tito RY , Joossens M , Raes J . Stool consistency is strongly associated with gut microbiota richness and composition, enterotypes and bacterial growth rates. Gut. 2016;65(1):57–62. 26069274 10.1136/gutjnl-2015-309618 PMC4717365

[45] Albenberg L , Esipova TV , Judge CP , Bittinger K , Chen J , Laughlin A , et al. Correlation between intraluminal oxygen gradient and radial partitioning of intestinal microbiota. Gastroenterology. 2014;147(5):1055–1063.e8. 25046162 10.1053/j.gastro.2014.07.020 PMC4252572

[46] Durbán A , Abellán JJ , Jiménez‐Hernández N , Ponce M , Ponce J , Sala T , et al. Assessing gut microbial diversity from feces and rectal mucosa. Microb Ecol. 2011;61(1):123–133. 20734040 10.1007/s00248-010-9738-y

[47] Weyrich LS , Farrer AG , Eisenhofer R , Arriola LA , Young J , Selway CA , et al. Laboratory contamination over time during low‐biomass sample analysis. Mol Ecol Resour. 2019;19(4):982–996. 30887686 10.1111/1755-0998.13011 PMC6850301

[48] Laurence M , Hatzis C , Brash DE . Common contaminants in next‐generation sequencing that hinder discovery of low‐abundance microbes. PLoS One. 2014;9(5):e97876. 24837716 10.1371/journal.pone.0097876 PMC4023998

[49] Bustin SA , Benes V , Garson JA , Hellemans J , Huggett J , Kubista M , et al. The MIQE guidelines: minimum information for publication of quantitative real‐time PCR experiments. Clin Chem. 2009;55(4):611–622. 19246619 10.1373/clinchem.2008.112797

[50] Flanagan L , Schmid J , Ebert M , Soucek P , Kunicka T , Liska V , et al. *Fusobacterium nucleatum* associates with stages of colorectal neoplasia development, colorectal cancer and disease outcome. Eur J Clin Microbiol Infect Dis. 2014;33(8):1381–1390. 24599709 10.1007/s10096-014-2081-3

[51] Choi H , Kim E , Kang J , Kim HJ , Lee JY , Choi J , et al. Real‐time PCR quantification of 9 periodontal pathogens in saliva samples from periodontally healthy Korean young adults. J Periodontal Implant Sci. 2018;48(4):261–271. 30202609 10.5051/jpis.2018.48.4.261 PMC6125667

[52] Kirakodu SS , Govindaswami M , Novak MJ , Ebersole JL , Novak KF . Optimizing qPCR for the quantification of periodontal pathogens in a complex plaque biofilm. Open Dent J. 2008;2:49–55. 19088882 10.2174/1874210600802010049 PMC2581537

[53] Xu M , Yamada M , Li M , Liu H , Chen SG , Han YW . FadA from *Fusobacterium nucleatum* utilizes both secreted and nonsecreted forms for functional oligomerization for attachment and invasion of host cells. J Biol Chem. 2007;282(34):25000–25009. 17588948 10.1074/jbc.M611567200

[54] Rubinstein MR , Wang X , Liu W , Hao Y , Cai G , Han YW . *Fusobacterium nucleatum* promotes colorectal carcinogenesis by modulating E‐cadherin/β‐catenin signaling via its FadA adhesin. Cell Host Microbe. 2013;14(2):195–206. 23954158 10.1016/j.chom.2013.07.012 PMC3770529

[55] Rubinstein MR , Baik JE , Lagana SM , Han RP , Raab WJ , Sahoo D , et al. *Fusobacterium nucleatum* promotes colorectal cancer by inducing Wnt/β‐catenin modulator Annexin A1. EMBO Rep. 2019;20(4):e47638. 30833345 10.15252/embr.201847638 PMC6446206

[56] Vasishtha S , Isenberg HD , Sood SK . *Gemella morbillorum* as a cause of septic shock. Clin Infect Dis. 1996;22(6):1084–1086. 8783715 10.1093/clinids/22.6.1084

[57] FitzGerald SF , Moloney AC , Maurer BJ , Hall WW . *Gemella* endocarditis: consider the colon. J Heart Valve Dis. 2006;15(6):833–835. 17152793

[58] Jiang Y , Qin W , Li J , Gao Y , Zeng Y . A case report of sepsis and death caused by *Parvimonas micra*, a rare anaerobe. Front Public Health. 2022;10:994279. 36203671 10.3389/fpubh.2022.994279 PMC9530780

[59] Xu J , Yang M , Wang D , Zhang S , Yan S , Zhu Y , et al. Alteration of the abundance of *Parvimonas micra* in the gut along the adenoma‐carcinoma sequence. Oncol Lett. 2020;20(4):106. 32831925 10.3892/ol.2020.11967 PMC7439112

[60] Bergsten E , Mestivier D , Donnadieu F , Pedron T , Tsoumtsa L , Lemichez E , et al. *Parvimonas micra*, an oral pathobiont associated with colorectal cancer, epigenetically reprograms human primary intestinal epithelial cells. bioRxiv. 2022. 10.1101/2022.05.14.491935 PMC10580862 37842920

[61] Löwenmark T , Li X , Löfgren‐Burström A , Zingmark C , Ling A , Kellgren TG , et al. *Parvimonas micra* is associated with tumour immune profiles in molecular subtypes of colorectal cancer. Cancer Immunol Immunother. 2022;71(10):2565–2575. 35301576 10.1007/s00262-022-03179-4 PMC9463256

[62] Kerdreux M , Edin S , Löwenmark T , Bronnec V , Löfgren‐Burström A , Zingmark C , et al. *Porphyromonas gingivalis* in colorectal cancer and its association to patient prognosis. J Cancer. 2023;14(9):1479–1485. 37325051 10.7150/jca.83395 PMC10266249

