# Supplementary material for: Detection of colorectal‐cancer‐associated bacterial taxa in fecal samples using next‐generation sequencing and 19 newly established qPCR assays
Source: Mol Oncol. 2024 Jul 6;19(2):412–29. doi: 10.1002/1878-0261.13700 (PMC11793011; doi:10.1002/1878-0261.13700)
Supplement: Supplementary file 1 — Fig. S1. Alpha diversity. Fig. S2. Beta diversity analysis did not reveal any significant differences in fecal microbial composition between the groups; cancer patients, patients with adenomatous polyps and healthy controls (Bray‐Curtis P = 0.95, UniFrac P = 0.78). [file MOL2-19-412-s003.docx]

Detection of colorectal cancer-associated bacterial taxa in fecal samples using next-generation sequencing and 19 newly established qPCR assays

Thulasika Senthakumaran^1^, Tone M. Tannæs^2,3^, Aina E. F. Moen^2,3,4^, Stephan A. Brackmann^5,6^, David Jahanlu^1^, Trine B. Rounge^7,8^, *Vahid Bemanian^9^, *Hege S. Tunsjø^1^

^1^Department of Life Sciences and Health, Oslo Metropolitan University, Oslo, Norway; ^2^Section for Clinical Molecular Biology (EpiGen), Akershus University Hospital, Lørenskog, Norway; ^3^Department of Clinical Molecular Biology, Institute of Clinical Medicine, University of Oslo, Oslo, Norway; ^4^Department of Methods Development and Analytics, Norwegian Institute of Public Health, Oslo, Norway; ^5^Department of Gastroenterology, Division of Medicine, Akershus University Hospital, Lørenskog, Norway; ^6^Institute for Clinical Medicine, University of Oslo, Oslo, Norway; ^7^Centre for Bioinformatics, Department of Pharmacy, University of Oslo, Oslo, Norway; ^8^Department of Research, Cancer Registry of Norway, Oslo, Norway; ^9^Department of Pathology, Akershus University Hospital, Lørenskog, Norway.

Supplementary figures: Alpha and beta diversity


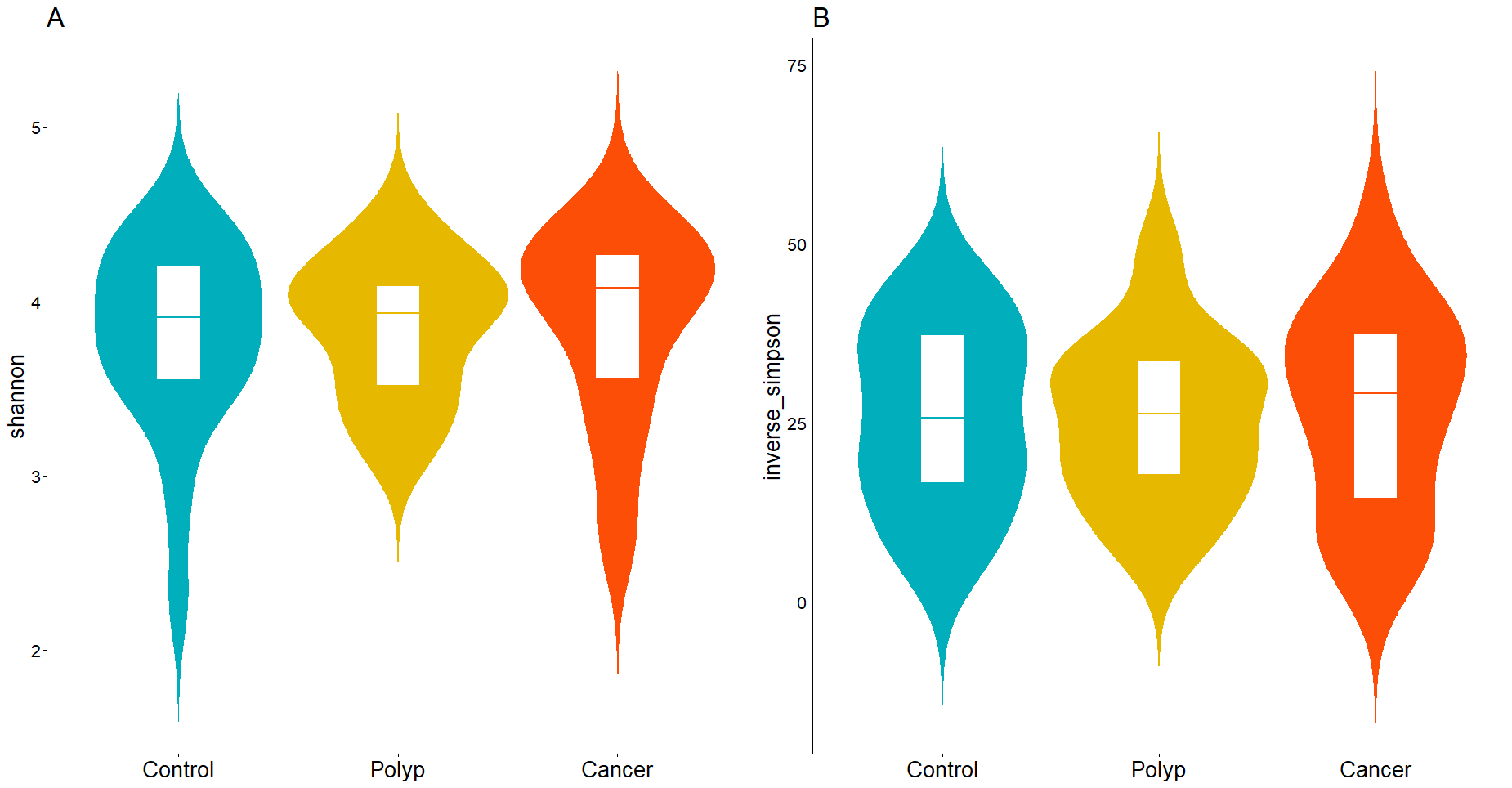


Supplementary figure 1: Alpha diversity. Violin plots with box plots illustrating Shannon and Inverse Simpson diversity indices in fecal samples from cancer patients, patients with adenomatous polyps (Polyp) and healthy controls. There are no significant differences in alpha diversity between the groups (p = 0.72 and 0.83 for Shannon and Inverse Simpson, respectively).


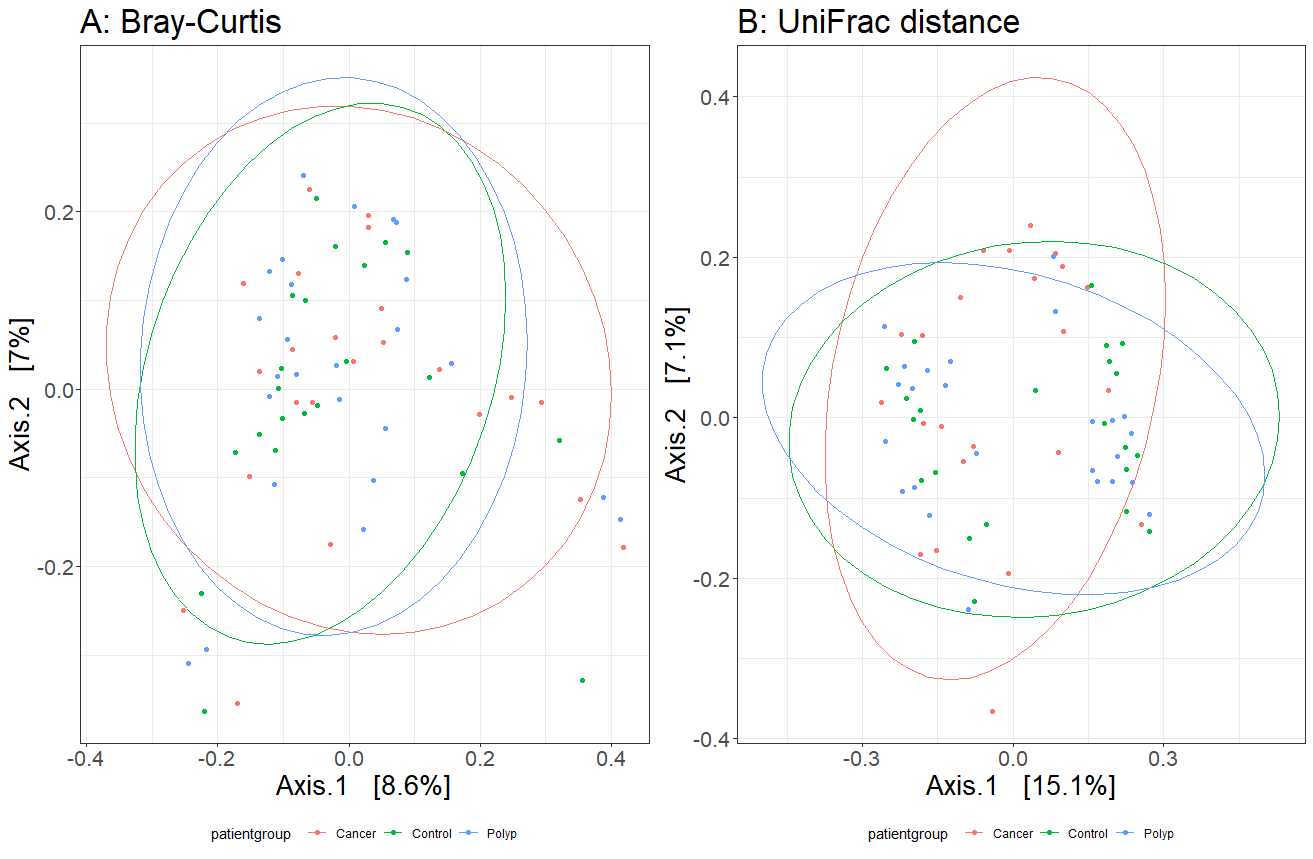


Supplementary figure 2: Beta diversity analysis did not reveal any significant differences in fecal microbial composition between the groups; cancer patients, patients with adenomatous polyps and healthy controls ( Bray-Curtis p = 0.95, UniFrac p = 0.78).
