# Supplementary material for: Detection of colorectal‐cancer‐associated bacterial taxa in fecal samples using next‐generation sequencing and 19 newly established qPCR assays
Source: Mol Oncol. 2024 Jul 6;19(2):412–29. doi: 10.1002/1878-0261.13700 (PMC11793011; doi:10.1002/1878-0261.13700)
Supplement: Supplementary file 2 — Table S1. Patient information and feature counts from fecal samples. [file MOL2-19-412-s002.docx]

Detection of colorectal cancer-associated bacterial taxa in fecal samples using next-generation sequencing and 19 newly established qPCR assays

Thulasika Senthakumaran^1^, Tone M. Tannæs^2,3^, Aina E. F. Moen^2,3,4^, Stephan A. Brackmann^5,6^, David Jahanlu^1^, Trine B. Rounge^7,8^, *Vahid Bemanian^9^, *Hege S. Tunsjø^1^

^1^Department of Life Sciences and Health, Oslo Metropolitan University, Oslo, Norway; ^2^Section for Clinical Molecular Biology (EpiGen), Akershus University Hospital, Lørenskog, Norway; ^3^Department of Clinical Molecular Biology, Institute of Clinical Medicine, University of Oslo, Oslo, Norway; ^4^Department of Methods Development and Analytics, Norwegian Institute of Public Health, Oslo, Norway; ^5^Department of Gastroenterology, Division of Medicine, Akershus University Hospital, Lørenskog, Norway; ^6^Institute for Clinical Medicine, University of Oslo, Oslo, Norway; ^7^Centre for Bioinformatics, Department of Pharmacy, University of Oslo, Oslo, Norway; ^8^Department of Research, Cancer Registry of Norway, Oslo, Norway; ^9^Department of Pathology, Akershus University Hospital, Lørenskog, Norway.

Supplementary Table 1: Patient information and feature counts from fecal samples

Supplementary Table 1a: Table with information of sex, age, tumor location, classification of tumor, number of feature counts and Shannon index for cancer patients

| **Sample ID** | **Gender** | **Age** | **Tumor Location** | **Tumor classification T N M** | | | **Feature Count** | **Shannon Index** |
| --- | --- | --- | --- | --- | --- | --- | --- | --- |
| C1 | M | 69 | Sigmoid colon | 2 | 0 | 0 | 41341 | 3.266 |
| C2 | M | 74 | Sigmoid colon | Haggit: level 2 | | | 43719 | 3.904 |
| C3 | F | 57 | Cecum | 2 | 0 | 0 | 36462 | 4.079 |
| C4 | F | 53 | Cecum | 3 | 2 | 0 | 46629 | 4.376 |
| C5 | M | 70 | Rectosigmoid junction | 3 | 2 | 0 | 40373 | 4.326 |
| C6 | F | 80 | Transverse colon | 3 | 1 | 0 | 46488 | 4.135 |
| C7 | M | 69 | Rectum | 2 | 0 | 0 | 42018 | 4.427 |
| C8 | M | 71 | Sigmoid colon | 2 | 0 | 0 | 29263 | 3.793 |
| C9 | M | 67 | Ascending colon | 3 | 1 | 0 | 15724 | 4.252 |
| C10 | M | 72 | Sigmoid colon | 2 | 0 | 0 | 45691 | 3.351 |
| C11 | F | 80 | Ascending colon | 3 | 0 | 0 | 8607 | 3.607 |
| C12 | M | 75 | Transverse colon | 1 | 0 | 0 | 45009 | 4.251 |
| C13 | M | 68 | Sigmoid colon | 3 | 0 | 1 | 40677 | 4.021 |
| C14 | M | 68 | Cecum | 2 | 0 | 0 | 38471 | 4.286 |
| C15 | M | 63 | Rectosigmoid junction | 3 | 0 | 0 | 34869 | 3.508 |
| C16 | M | 84 | Cecum | 4 | 2 | 1 | 33623 | 3.046 |
| C17 | M | 76 | Ascending colon | 3 | 2 | 0 | 26616 | 3.942 |
| C18 | M | 65 | Sigmoid colon | 1 | 0 | 0 | 49642 | 2.628 |
| C19 | F | 70 | Cecum | 2 | 0 | 0 | 32746 | 4.117 |
| C20 | F | 55 | Cecum | 4 | 1 | 1 | 35856 | 4.148 |
| C21 | M | 45 | Sigmoid colon | 4 | 1 | 1 | - | - |
| C22 | M | 66 | Sigmoid colon | 3 | 0 | 0 | 46308 | 2.647 |
| C23 | M | 82 | Rectum | 2 | 0 | 0 | 40938 | 4.359 |
| C24 | M | 81 | Rectum | 2 | 0 | 0 | 40396 | 4.557 |
| C25 | F | 73 | Sigmoid colon | 4b | 1b | 0 | - | - |

Supplementary Table 1b: Table with information of sex, age, number of feature counts and Shannon index for adenomatous polyp patients

| **Sample ID** | **Gender** | **Age** | **Feature Count** | **Shannon Index** |
| --- | --- | --- | --- | --- |
| P1 | M | 51 | 43852 | 3.936 |
| P2 | M | 69 | 36110 | 4.438 |
| P3 | F | 82 | 40789 | 3.422 |
| P4 | F | 59 | 46725 | 3.206 |
| P5 | F | 72 | 62359 | 3.079 |
| P6 | M | 66 | 40121 | 4.246 |
| P7 | F | 80 | 47035 | 4.295 |
| P8 | M | 63 | 39476 | 3.196 |
| P9 | M | 56 | 44183 | 3.476 |
| P10 | F | 61 | 40625 | 4.088 |
| P11 | F | 55 | 41810 | 3.521 |
| P12 | M | 75 | 51433 | 3.371 |
| P13 | F | 71 | 43137 | 3.803 |
| P14 | M | 50 | 45885 | 3.893 |
| P15 | F | 80 | 38862 | 4.130 |
| P16 | F | 52 | 52868 | 4.514 |
| P17 | M | 70 | 41327 | 4.090 |
| P18 | F | 69 | 41516 | 3.714 |
| P19 | F | 82 | 49954 | 4.050 |
| P20 | M | 74 | 32624 | 3.935 |
| P21 | M | 78 | 44901 | 3.933 |
| P22 | F | 55 | 35870 | 4.094 |
| P23 | F | 70 | 36982 | 4.280 |
| P24 | M | 64 | 32812 | 4.027 |
| P25 | F | 68 | 33569 | 3.582 |

Supplementary Table 1c: Table with information of sex, age, number of feature counts and Shannon index for healthy controls

| **Sample ID** | **Gender** | **Age** | **Feature Count** | **Shannon Index** |
| --- | --- | --- | --- | --- |
| K1 | M | 65 | 59471 | 3.999 |
| K2 | F | 59 | 51876 | 4.070 |
| K3 | M | 52 | 48221 | 3.502 |
| K4 | M | 64 | 26054 | 2.841 |
| K5 | M | 81 | 44156 | 4.489 |
| K6 | F | 52 | 69579 | 2.295 |
| K7 | F | 41 | 46411 | 3.684 |
| K8 | F | 75 | 36998 | 3.193 |
| K9 | M | 68 | 39911 | 3.929 |
| K10 | M | 75 | 38244 | 3.740 |
| K11 | M | 30 | 21132 | 3.893 |
| K12 | M | 51 | 48442 | 3.433 |
| K13 | F | 67 | 44960 | 3.928 |
| K14 | F | 62 | 42273 | 4.303 |
| K15 | M | 58 | 41915 | 4.216 |
| K16 | F | 40 | 50177 | 3.678 |
| K17 | M | 49 | 53331 | 3.544 |
| K18 | M | 87 | 53065 | 4.480 |
| K19 | F | 46 | 41957 | 4.340 |
| K20 | F | 52 | 49413 | 4.165 |
| K21 | M | 51 | 56614 | 3.590 |
| K22 | M | 61 | 50633 | 4.334 |
