# Supplementary material for: Detection of colorectal‐cancer‐associated bacterial taxa in fecal samples using next‐generation sequencing and 19 newly established qPCR assays
Source: Mol Oncol. 2024 Jul 6;19(2):412–29. doi: 10.1002/1878-0261.13700 (PMC11793011; doi:10.1002/1878-0261.13700)
Supplement: Supplementary file 3 — Table S2. Reference sequence. [file MOL2-19-412-s004.docx]

Detection of colorectal cancer-associated bacterial taxa in fecal samples using next-generation sequencing and 19 newly established qPCR assays

Thulasika Senthakumaran^1^, Tone M. Tannæs^2,3^, Aina E. F. Moen^2,3,4^, Stephan A. Brackmann^5,6^, David Jahanlu^1^, Trine B. Rounge^7,8^, *Vahid Bemanian^9^, *Hege S. Tunsjø^1^

^1^Department of Life Sciences and Health, Oslo Metropolitan University, Oslo, Norway; ^2^Section for Clinical Molecular Biology (EpiGen), Akershus University Hospital, Lørenskog, Norway; ^3^Department of Clinical Molecular Biology, Institute of Clinical Medicine, University of Oslo, Oslo, Norway; ^4^Department of Methods Development and Analytics, Norwegian Institute of Public Health, Oslo, Norway; ^5^Department of Gastroenterology, Division of Medicine, Akershus University Hospital, Lørenskog, Norway; ^6^Institute for Clinical Medicine, University of Oslo, Oslo, Norway; ^7^Centre for Bioinformatics, Department of Pharmacy, University of Oslo, Oslo, Norway; ^8^Department of Research, Cancer Registry of Norway, Oslo, Norway; ^9^Department of Pathology, Akershus University Hospital, Lørenskog, Norway.

Supplementary Table 2: Reference sequence

Supplementary Table 2: GenBank accession number of the references sequences used to design primers and probes

| **Species of interest** | **GenBank accession no.** |
| --- | --- |
| *Fusobacterium nucleatum ssp* | CP028101.1 |
| *Fusobacterium periodonticum* | CP028108.1 |
| *Fusobacterium gonidiaformans* | CP028106.1 |
| *Gemella morbillorum* | CP046314.1 |
| *Gemella haemolysans* | HM103930.1 |
| *Gemella sanguinis* | AB775591.1 |
| *Leptotrichia trevisanii* | AY029805.1 |
| *Leptotrichia hofstadii* | NR_025647.1 |
| *Leptotrichia shahii* | NR_025648.1 |
| *Leptotrichia wadei* | NR_036844.1 |
| *Leptotrichia goodfellowii* | AY029807.1 |
| *Granulicatella adiacens* | CP102283.1 |
| *Porphyromonas gingivalis* | NZ_CP116614.1 |
| *Porphyromonas asaccharolytica* | NZ_AENO01000051.1 |
| *Porphyromonas endodontalis* | NZ_ACNN01000036.1 |
| *Prevotella copri* | CP102288.1 |
| *Campylobacter concisus* | NZ_CP012541.1 |
| *Peptostreptococcus stomatis* | MT328661.1 |
