# Supplementary material for: Detection of colorectal‐cancer‐associated bacterial taxa in fecal samples using next‐generation sequencing and 19 newly established qPCR assays
Source: Mol Oncol. 2024 Jul 6;19(2):412–29. doi: 10.1002/1878-0261.13700 (PMC11793011; doi:10.1002/1878-0261.13700)
Supplement: Supplementary file 4 — Table S3. Analytical specificity. [file MOL2-19-412-s001.docx]

Detection of colorectal cancer-associated bacterial taxa in fecal samples using next-generation sequencing and 19 newly established qPCR assays

Thulasika Senthakumaran^1^, Tone M. Tannæs^2,3^, Aina E. F. Moen^2,3,4^, Stephan A. Brackmann^5,6^, David Jahanlu^1^, Trine B. Rounge^7,8^, *Vahid Bemanian^9^, *Hege S. Tunsjø^1^

^1^Department of Life Sciences and Health, Oslo Metropolitan University, Oslo, Norway; ^2^Section for Clinical Molecular Biology (EpiGen), Akershus University Hospital, Lørenskog, Norway; ^3^Department of Clinical Molecular Biology, Institute of Clinical Medicine, University of Oslo, Oslo, Norway; ^4^Department of Methods Development and Analytics, Norwegian Institute of Public Health, Oslo, Norway; ^5^Department of Gastroenterology, Division of Medicine, Akershus University Hospital, Lørenskog, Norway; ^6^Institute for Clinical Medicine, University of Oslo, Oslo, Norway; ^7^Centre for Bioinformatics, Department of Pharmacy, University of Oslo, Oslo, Norway; ^8^Department of Research, Cancer Registry of Norway, Oslo, Norway; ^9^Department of Pathology, Akershus University Hospital, Lørenskog, Norway.

Supplementary Table 3: Analytical specificity

Supplementary Table 3: Analytical specificity

|  |  | ***F.nucleatum ssp*** | ***F. periodonticum*** | ***F. gonidiaformans*** | ***P. micra*** | ***G. morbillorum*** | ***G. haemolysans*** | ***G. sanguinis*** | ***L. trevisanii*** | ***L. hofstadii*** | ***L. shahii*** |
| --- | --- | --- | --- | --- | --- | --- | --- | --- | --- | --- | --- |
| Fusobacterium gonidiaforum | Clinical isolate | Undetermined | Undetermined | 23,681 | Undetermined | Undetermined | Undetermined | Undetermined | Undetermined | Undetermined | Undetermined |
| Fusobacterium necrophorum | CCUG 59679 | Undetermined | Undetermined | 26,289 | Undetermined | Undetermined | Undetermined | Undetermined | Undetermined | Undetermined | Undetermined |
| Fusobacterium periodonticum | CCUG 51780 | Undetermined | 24.99 | Undetermined | Undetermined | Undetermined | Undetermined | Undetermined | Undetermined | Undetermined | Undetermined |
| Fusobacterium nucleatum polymorpum | CCUG 60920 | 23,03 | Undetermined | Undetermined | Undetermined | Undetermined | Undetermined | Undetermined | Undetermined | Undetermined | Undetermined |
| Fusobacterium nucleatum animalis | CCUG 32879T | 20,67 | Undetermined | Undetermined | Undetermined | Undetermined | Undetermined | Undetermined | Undetermined | Undetermined | Undetermined |
| Fusobacterium nucleatum vincentii | CCUG 37843T | 22,43 | Undetermined | Undetermined | Undetermined | Undetermined | Undetermined | Undetermined | Undetermined | Undetermined | Undetermined |
| Fusobacterium nucleatum nucleatum | CCUG 33059T | 22,05 | Undetermined | Undetermined | Undetermined | Undetermined | Undetermined | Undetermined | Undetermined | Undetermined | Undetermined |
| Fusobacterium varium | CCUG 4858T | Undetermined | Undetermined | Undetermined | Undetermined | Undetermined | Undetermined | Undetermined | Undetermined | Undetermined | Undetermined |
| Fusobacterium naviforme | CCUG 50052T | Undetermined | Undetermined | Undetermined | Undetermined | Undetermined | Undetermined | Undetermined | Undetermined | Undetermined | Undetermined |
| Fusobacterium mortiferum | Clinical isolate | Undetermined | Undetermined | Undetermined | Undetermined | Undetermined | 37,850 | Undetermined | Undetermined | Undetermined | Undetermined |
| Gemella morbillorum | Clinical isolate | Undetermined | Undetermined | Undetermined | Undetermined | 24,64 | 36,921 | Undetermined | Undetermined | Undetermined | Undetermined |
| Gemella haemolysans | Clinical isolate | Undetermined | Undetermined | Undetermined | Undetermined | Undetermined | 31,043 | 36,284 | Undetermined | Undetermined | Undetermined |
| Gemella bergeri | Clinical isolate | Undetermined | Undetermined | 37,376 | Undetermined | Undetermined | Undetermined | 35,068 | Undetermined | Undetermined | Undetermined |
| Gemella sanguinis | CCUG 37820T | Undetermined | Undetermined | Undetermined | Undetermined | Undetermined | Undetermined | 21,742 | Undetermined | Undetermined | Undetermined |
| Porphyromonas gingivalis | CCUG 25893T | Undetermined | Undetermined | Undetermined | Undetermined | Undetermined | Undetermined | Undetermined | Undetermined | Undetermined | Undetermined |
| Porphyromonas asaccharolytica | Clinical isolate | Undetermined | Undetermined | Undetermined | Undetermined | Undetermined | Undetermined | Undetermined | Undetermined | Undetermined | Undetermined |
| Peptostreptococcus anerobius | CCUG 7835T | Undetermined | Undetermined | Undetermined | Undetermined | Undetermined | Undetermined | Undetermined | Undetermined | Undetermined | Undetermined |
| Peptostreptococcus stomatis | CCUG 51858T | Undetermined | Undetermined | Undetermined | Undetermined | Undetermined | 37,272 | Undetermined | Undetermined | Undetermined | Undetermined |
| Parvimonas micra | CCUG 46357T | Undetermined | Undetermined | Undetermined | 28,99 | Undetermined | 36,898 | Undetermined | Undetermined | Undetermined | Undetermined |
| Leptrotrichia goodfellowii | CCUG 53232 | Undetermined | Undetermined | Undetermined | Undetermined | Undetermined | Undetermined | Undetermined | Undetermined | Undetermined | Undetermined |
| Leptrotrichia shahii | CCUG 47503T | Undetermined | Undetermined | Undetermined | Undetermined | Undetermined | Undetermined | Undetermined | Undetermined | 31,568 | 23,991 |
| Leptrotrichia trevisanii | CCUG 72923 | Undetermined | Undetermined | Undetermined | Undetermined | Undetermined | Undetermined | Undetermined | 26,282 | Undetermined | Undetermined |
| Leptrotrichia hofstadii | CCUG 47504T | Undetermined | Undetermined | Undetermined | Undetermined | Undetermined | Undetermined | Undetermined | Undetermined | 26,687 | 32,859 |
| Leptrotrichia wadei | CCUG 63608 | Undetermined | Undetermined | Undetermined | Undetermined | Undetermined | Undetermined | Undetermined | Undetermined | Undetermined | Undetermined |
| Granulicatella adiacens | Clinical isolate | Undetermined | Undetermined | Undetermined | Undetermined | Undetermined | Undetermined | Undetermined | Undetermined | Undetermined | Undetermined |
| Granulicatella elegans | Clinical isolate | Undetermined | Undetermined | Undetermined | Undetermined | Undetermined | Undetermined | Undetermined | Undetermined | Undetermined | Undetermined |
| Hungatella hathewayi | CCUG 43506T | Undetermined | Undetermined | Undetermined | Undetermined | Undetermined | Undetermined | Undetermined | Undetermined | Undetermined | Undetermined |
| Blautia marasmi | CCUG 70622T | Undetermined | Undetermined | Undetermined | Undetermined | Undetermined | Undetermined | Undetermined | Undetermined | Undetermined | Undetermined |
| Escherichia coli | CCUG 25922 | Undetermined | Undetermined | Undetermined | Undetermined | Undetermined | Undetermined | Undetermined | Undetermined | Undetermined | Undetermined |
| Filifactor alocis | CCUG 47790T | Undetermined | Undetermined | Undetermined | Undetermined | Undetermined | Undetermined | Undetermined | Undetermined | Undetermined | Undetermined |
| Streptococcus mutans | CCUG 69401 | Undetermined | Undetermined | Undetermined | Undetermined | Undetermined | 39,094 | Undetermined | Undetermined | Undetermined | Undetermined |
| Streptococcus mitis | CCUG 70037 | Undetermined | Undetermined | Undetermined | Undetermined | Undetermined | Undetermined | Undetermined | Undetermined | Undetermined | Undetermined |
| Streptococcus aureus | ATCC 6538 | Undetermined | Undetermined | Undetermined | Undetermined | Undetermined | Undetermined | Undetermined | Undetermined | Undetermined | Undetermined |
| Streptococcus oralis | CCUG 67092 | Undetermined | Undetermined | Undetermined | Undetermined | Undetermined | Undetermined | Undetermined | Undetermined | Undetermined | Undetermined |
| Streptococcus epidermis | CCUG 37337 | Undetermined | Undetermined | Undetermined | Undetermined | Undetermined | 37,182 | Undetermined | Undetermined | Undetermined | Undetermined |
| Citrobacter freunalii | Clinical isolate | Undetermined | Undetermined | Undetermined | Undetermined | Undetermined | 39,144 | Undetermined | Undetermined | Undetermined | Undetermined |
| Klebsiella pneumoniae | CCUG 45421 | Undetermined | Undetermined | Undetermined | Undetermined | Undetermined | Undetermined | Undetermined | Undetermined | Undetermined | Undetermined |
| Anaerococcus vaginalis | CCUG 31349T | Undetermined | Undetermined | Undetermined | Undetermined | Undetermined | 38,091 | Undetermined | Undetermined | Undetermined | Undetermined |
| Clostridium perfringens | Clinical isolate | Undetermined | Undetermined | Undetermined | Undetermined | Undetermined | 37,082 | Undetermined | Undetermined | Undetermined | Undetermined |
| Clostridium difficile | CCUG 54206 | Undetermined | Undetermined | Undetermined | Undetermined | Undetermined | Undetermined | Undetermined | Undetermined | Undetermined | Undetermined |
| Clostridium septicum | Clinical isolate | Undetermined | Undetermined | Undetermined | Undetermined | Undetermined | Undetermined | Undetermined | Undetermined | Undetermined | Undetermined |
| Bacteroides thetaiotamicron | Clinical isolate | Undetermined | Undetermined | Undetermined | Undetermined | Undetermined | Undetermined | Undetermined | Undetermined | Undetermined | Undetermined |
| Bacteriodes fragilis | Clinical isolate | Undetermined | Undetermined | Undetermined | Undetermined | Undetermined | Undetermined | Undetermined | Undetermined | Undetermined | Undetermined |
| Bifidobacterium longum | Clinical isolate | Undetermined | Undetermined | Undetermined | Undetermined | Undetermined | 37,116 | Undetermined | Undetermined | Undetermined | Undetermined |
| Bifidobacterium longum ssp longum | CCUG 28903T | Undetermined | Undetermined | Undetermined | Undetermined | Undetermined | Undetermined | Undetermined | Undetermined | Undetermined | Undetermined |
| Psedomonas aerugi | CCUG 59347 | Undetermined | Undetermined | Undetermined | Undetermined | Undetermined | 38,191 | Undetermined | Undetermined | Undetermined | Undetermined |
| Enterococcus faecalis | ATCC 29212 | Undetermined | Undetermined | Undetermined | Undetermined | Undetermined | Undetermined | Undetermined | Undetermined | Undetermined | Undetermined |
| Enterobacter cloacae | CCUG 33986 | Undetermined | Undetermined | Undetermined | Undetermined | Undetermined | Undetermined | Undetermined | Undetermined | Undetermined | Undetermined |
| Phascolarctobacterium faecium | DSM 14760 | Undetermined | Undetermined | Undetermined | Undetermined | Undetermined | Undetermined | Undetermined | Undetermined | Undetermined | Undetermined |
| Phascolarctobacterium succinatutens | DSM 22533 | Undetermined | Undetermined | Undetermined | Undetermined | Undetermined | 38,145 | Undetermined | Undetermined | Undetermined | Undetermined |
| Campylobacter concisus | CCUG 13144T | - | - | - | - | - | - | - | - | - | - |
| Porphyromonas endodontalis | CCUG 16442T | - | - | - | - | - | - | - | - | - | - |
| Prevotella copri | CCUG 58058T | - | - | - | - | - | - | - | - | - | - |

Supplementary Table 3 continued: Analytical specificity

|  |  | ***L. wadei*** | ***L. goodfellowii*** | ***G. adiasens/ G. elegans*** | ***G. adiasens*** | ***P. gingivalis*** | ***P. asaccharolytica*** | ***P. endodontalis*** | ***P. copri*** | ***C. consisus*** | ***P. stomatitis*** |
| --- | --- | --- | --- | --- | --- | --- | --- | --- | --- | --- | --- |
| Fusobacterium gonidiaforum | Clinical isolate | Undetermined | Undetermined | Undetermined | Undetermined | Undetermined | Undetermined | Undetermined | Undetermined | Undetermined | Undetermined |
| Fusobacterium necrophorum | CCUG 59679 | Undetermined | Undetermined | Undetermined | Undetermined | Undetermined | Undetermined | Undetermined | Undetermined | Undetermined | Undetermined |
| Fusobacterium periodonticum | CCUG 51780 | Undetermined | Undetermined | Undetermined | Undetermined | Undetermined | Undetermined | Undetermined | Undetermined | Undetermined | Undetermined |
| Fusobacterium nucleatum polymorpum | CCUG 60920 | Undetermined | Undetermined | Undetermined | Undetermined | Undetermined | Undetermined | Undetermined | Undetermined | Undetermined | Undetermined |
| Fusobacterium nucleatum animalis | CCUG 32879T | Undetermined | Undetermined | Undetermined | Undetermined | Undetermined | Undetermined | Undetermined | Undetermined | Undetermined | Undetermined |
| Fusobacterium nucleatum vincentii | CCUG 37843T | Undetermined | Undetermined | Undetermined | Undetermined | Undetermined | Undetermined | Undetermined | Undetermined | Undetermined | Undetermined |
| Fusobacterium nucleatum nucleatum | CCUG 33059T | Undetermined | Undetermined | Undetermined | Undetermined | Undetermined | Undetermined | Undetermined | Undetermined | Undetermined | Undetermined |
| Fusobacterium varium | CCUG 4858T | Undetermined | Undetermined | Undetermined | Undetermined | Undetermined | Undetermined | Undetermined | Undetermined | Undetermined | Undetermined |
| Fusobacterium naviforme | CCUG 50052T | Undetermined | Undetermined | Undetermined | Undetermined | Undetermined | 39,480 | Undetermined | Undetermined | Undetermined | Undetermined |
| Fusobacterium mortiferum | Clinical isolate | Undetermined | Undetermined | Undetermined | Undetermined | Undetermined | Undetermined | Undetermined | 39,332 | Undetermined | Undetermined |
| Gemella morbillorum | Clinical isolate | Undetermined | Undetermined | Undetermined | Undetermined | Undetermined | Undetermined | Undetermined | Undetermined | Undetermined | Undetermined |
| Gemella haemolysans | Clinical isolate | Undetermined | Undetermined | Undetermined | Undetermined | Undetermined | Undetermined | Undetermined | Undetermined | Undetermined | Undetermined |
| Gemella bergeri | Clinical isolate | Undetermined | Undetermined | Undetermined | Undetermined | Undetermined | Undetermined | Undetermined | Undetermined | Undetermined | Undetermined |
| Gemella sanguinis | CCUG 37820T | Undetermined | Undetermined | Undetermined | Undetermined | Undetermined | Undetermined | Undetermined | Undetermined | Undetermined | Undetermined |
| Porphyromonas gingivalis | CCUG 25893T | Undetermined | Undetermined | Undetermined | Undetermined | 27,029 | Undetermined | Undetermined | Undetermined | Undetermined | Undetermined |
| Porphyromonas asaccharolytica | Clinical isolate | Undetermined | Undetermined | Undetermined | Undetermined | Undetermined | 25,146 | Undetermined | Undetermined | Undetermined | Undetermined |
| Peptostreptococcus anerobius | CCUG 7835T | Undetermined | Undetermined | Undetermined | Undetermined | Undetermined | Undetermined | Undetermined | 38,457 | Undetermined | Undetermined |
| Peptostreptococcus stomatis | CCUG 51858T | Undetermined | Undetermined | Undetermined | Undetermined | 38,426 | Undetermined | Undetermined | Undetermined | Undetermined | 28,397 |
| Parvimonas micra | CCUG 46357T | Undetermined | Undetermined | Undetermined | Undetermined | Undetermined | Undetermined | Undetermined | Undetermined | Undetermined | Undetermined |
| Leptrotrichia goodfellowii | CCUG 53232 | Undetermined | 25,586 | Undetermined | Undetermined | Undetermined | Undetermined | Undetermined | Undetermined | Undetermined | Undetermined |
| Leptrotrichia shahii | CCUG 47503T | Undetermined | Undetermined | Undetermined | Undetermined | Undetermined | Undetermined | Undetermined | Undetermined | Undetermined | Undetermined |
| Leptrotrichia trevisanii | CCUG 72923 | Undetermined | Undetermined | Undetermined | Undetermined | Undetermined | Undetermined | Undetermined | Undetermined | Undetermined | Undetermined |
| Leptrotrichia hofstadii | CCUG 47504T | Undetermined | Undetermined | Undetermined | Undetermined | Undetermined | Undetermined | Undetermined | Undetermined | Undetermined | Undetermined |
| Leptrotrichia wadei | CCUG 63608 | 24,801 | Undetermined | Undetermined | Undetermined | Undetermined | Undetermined | Undetermined | Undetermined | Undetermined | Undetermined |
| Granulicatella adiacens | Clinical isolate | Undetermined | Undetermined | 26,406 | 28,007 | Undetermined | Undetermined | Undetermined | Undetermined | Undetermined | Undetermined |
| Granulicatella elegans | Clinical isolate | Undetermined | Undetermined | 27,262 | Undetermined | Undetermined | Undetermined | Undetermined | Undetermined | Undetermined | Undetermined |
| Hungatella hathewayi | CCUG 43506T | Undetermined | Undetermined | Undetermined | Undetermined | Undetermined | Undetermined | Undetermined | Undetermined | Undetermined | Undetermined |
| Blautia marasmi | CCUG 70622T | Undetermined | Undetermined | Undetermined | Undetermined | Undetermined | Undetermined | Undetermined | 37,866 | Undetermined | Undetermined |
| Escherichia coli | CCUG 25922 | Undetermined | Undetermined | Undetermined | Undetermined | Undetermined | Undetermined | Undetermined | Undetermined | Undetermined | Undetermined |
| Filifactor alocis | CCUG 47790T | Undetermined | Undetermined | Undetermined | Undetermined | Undetermined | Undetermined | Undetermined | Undetermined | Undetermined | Undetermined |
| Streptococcus mutans | CCUG 69401 | Undetermined | Undetermined | Undetermined | Undetermined | Undetermined | Undetermined | Undetermined | Undetermined | Undetermined | Undetermined |
| Streptococcus mitis | CCUG 70037 | Undetermined | Undetermined | Undetermined | Undetermined | Undetermined | Undetermined | Undetermined | 38,844 | Undetermined | Undetermined |
| Streptococcus aureus | ATCC 6538 | Undetermined | Undetermined | Undetermined | Undetermined | Undetermined | Undetermined | Undetermined | Undetermined | Undetermined | Undetermined |
| Streptococcus oralis | CCUG 67092 | Undetermined | Undetermined | Undetermined | Undetermined | Undetermined | Undetermined | Undetermined | Undetermined | Undetermined | Undetermined |
| Streptococcus epidermis | CCUG 37337 | Undetermined | Undetermined | Undetermined | Undetermined | Undetermined | Undetermined | Undetermined | Undetermined | Undetermined | Undetermined |
| Citrobacter freunalii | Clinical isolate | Undetermined | Undetermined | Undetermined | Undetermined | Undetermined | Undetermined | Undetermined | 38,837 | Undetermined | Undetermined |
| Klebsiella pneumoniae | CCUG 45421 | Undetermined | Undetermined | Undetermined | Undetermined | Undetermined | Undetermined | Undetermined | Undetermined | Undetermined | Undetermined |
| Anaerococcus vaginalis | CCUG 31349T | Undetermined | Undetermined | Undetermined | Undetermined | Undetermined | Undetermined | Undetermined | Undetermined | Undetermined | Undetermined |
| Clostridium perfringens | Clinical isolate | Undetermined | Undetermined | Undetermined | Undetermined | Undetermined | Undetermined | Undetermined | Undetermined | Undetermined | Undetermined |
| Clostridium difficile | CCUG 54206 | Undetermined | Undetermined | Undetermined | Undetermined | Undetermined | Undetermined | Undetermined | Undetermined | Undetermined | Undetermined |
| Clostridium septicum | Clinical isolate | Undetermined | Undetermined | Undetermined | Undetermined | Undetermined | Undetermined | Undetermined | Undetermined | Undetermined | Undetermined |
| Bacteroides thetaiotamicron | Clinical isolate | Undetermined | Undetermined | Undetermined | Undetermined | Undetermined | Undetermined | Undetermined | Undetermined | Undetermined | Undetermined |
| Bacteriodes fragilis | Clinical isolate | Undetermined | Undetermined | Undetermined | Undetermined | Undetermined | Undetermined | Undetermined | Undetermined | Undetermined | Undetermined |
| Bifidobacterium longum | Clinical isolate | Undetermined | Undetermined | Undetermined | Undetermined | Undetermined | Undetermined | Undetermined | Undetermined | Undetermined | Undetermined |
| Bifidobacterium longum ssp longum | CCUG 28903T | Undetermined | Undetermined | Undetermined | Undetermined | Undetermined | Undetermined | Undetermined | Undetermined | Undetermined | Undetermined |
| Psedomonas aerugi | CCUG 59347 | Undetermined | Undetermined | Undetermined | Undetermined | Undetermined | Undetermined | Undetermined | Undetermined | Undetermined | Undetermined |
| Enterococcus faecalis | ATCC 29212 | Undetermined | Undetermined | Undetermined | Undetermined | Undetermined | Undetermined | Undetermined | Undetermined | Undetermined | Undetermined |
| Enterobacter cloacae | CCUG 33986 | Undetermined | Undetermined | Undetermined | Undetermined | Undetermined | Undetermined | Undetermined | Undetermined | Undetermined | Undetermined |
| Phascolarctobacterium faecium | DSM 14760 | Undetermined | Undetermined | Undetermined | Undetermined | Undetermined | Undetermined | Undetermined | Undetermined | Undetermined | Undetermined |
| Phascolarctobacterium succinatutens | DSM 22533 | Undetermined | Undetermined | Undetermined | Undetermined | Undetermined | Undetermined | Undetermined | Undetermined | Undetermined | Undetermined |
| Campylobacter concisus | CCUG 13144T | - | - | - | - | - | - | - | - | 23,834 | - |
| Porphyromonas endodontalis | CCUG 16442T | - | - | - | - | - | - | 31,286 | - | - | - |
| Prevotella copri | CCUG 58058T | - | - | - | - | - | - | - | 25,850 | - | - |
